# HSPA8 orchestrates SNARE complex assembly to drive extracellular vesicle-mediated spread of p-tau217 in Alzheimer's disease

**DOI:** 10.1186/s40035-026-00570-4

**Published:** 2026-08-27

**Authors:** Bin Xu, Zhen Guo, Jun Chen, Yazhou Xie, Pan Wang, Xiao Xiong, Jiayi Yu, Zhi Xu, Yuan Fu, Zhuoqing Lan, Guoping Peng, Jing Zhang

**Affiliations:** 1 Department of Pathology, The First Affiliated Hospital, Zhejiang University School of Medicine, Hangzhou, Zhejiang 310003 China; 2 Nanhu Brain-computer Interface Institute, Hangzhou, Zhejiang 310002 China; 3 Lingang Laboratory, Shanghai, 200120 China; 4 School of Nursing and Health, Henan Medical College, Zhengzhou University, Zhengzhou, Henan 450001 China; 5 School of Brain Science and Brain Medicine, Zhejiang University, Hangzhou, Zhejiang 310012 China; 6 Department of Neurology, The First Affiliated Hospital, Zhejiang University School of Medicine, Hangzhou, Zhejiang, 310003 China; 7 National Human Brain Bank for Health and Disease, Zhejiang University, Hangzhou, Zhejiang 310012 China

**Keywords:** Alzheimer’s disease, Extracellular vesicles, p-tau217, HSPA8, SNARE complex

## Abstract

**Background:**

Dysregulation of multivesicular bodies (MVBs) in Alzheimer’s disease (AD) contributes to aberrant tau secretion via extracellular vesicles (EVs). This may potentially explain our previous paradoxical observation of elevated free-form p-tau217 alongside reduced p-tau217^+^ EVs in plasma. This study aimed to investigate the mechanisms underlying the reduction of p-tau217^+^ EVs to uncover AD therapeutic targets.

**Methods:**

By integrating hippocampal spatial transcriptomics of human brain with EV proteomics of cerebrospinal fluid, we identified key regulators of p-tau217^+^ EV release. Subsequently, we investigated the mechanisms underlying the synthesis and secretion of p-tau217^+^ EVs. The regulatory roles of these candidate proteins were systematically evaluated through shRNA knockdown and interference with a synthetic peptide in both Aβ_42_-treated cells and AD model mice.

**Results:**

Heat shock protein family A member 8 (HSPA8) was identified as a crucial regulator of EV biogenesis and release, mediating the Aβ–SNAP29 interaction to disrupt SNARE complex assembly and impair p-tau217^+^ EV secretion. In AD models, HSPA8 inhibition with shRNA rescued p-tau217^+^ EVs and improved cognitive function. Additionally, blocking the Aβ–SNAP29 interaction with a selective peptide inhibitor for HSPA8 reversed the decline in p-tau217^+^ EV and cognitive deficits.

**Conclusions:**

These findings reveal a role of HSPA8 in regulating the MVB-mediated EV release and tau propagation, and highlight HSPA8 as a promising therapeutic target for modifying AD progression.

**Graphical abstract:**

**Figure:**
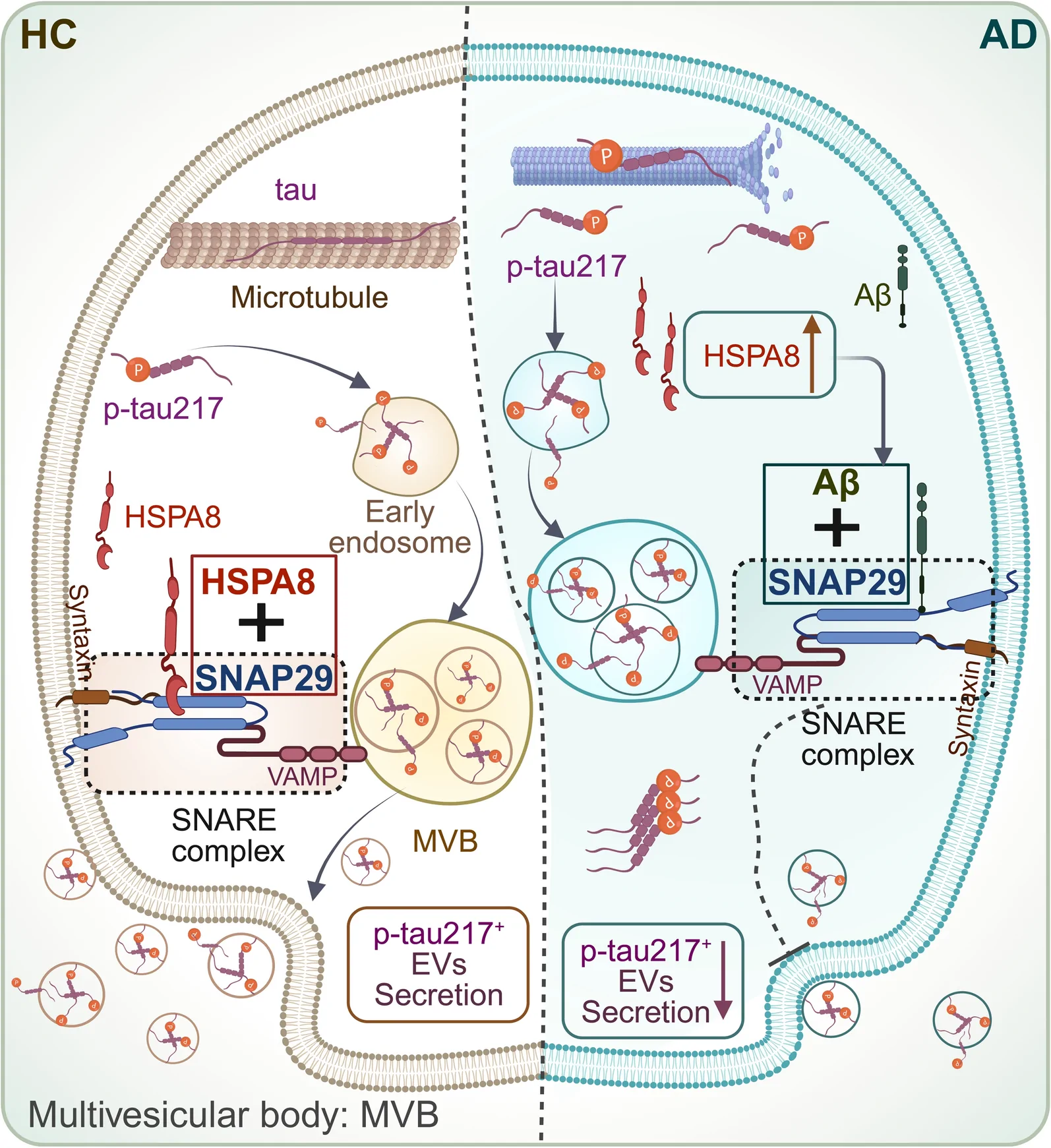

**Supplementary Information:**

The online version contains supplementary material available at 10.1186/s40035-026-00570-4.

## Background

Alzheimer’s disease (AD) is the most common neurodegenerative disease characterized clinically by progressive cognitive impairment [1, 2] and pathologically by amyloid-beta (Aβ) plaques and hyperphosphorylated neurofibrillary tau tangles [3]. While Aβ deposition has long been considered a key pathological hallmark, increasing evidence indicates that tau pathology correlates more closely with neuronal dysfunction and cognitive impairment, highlighting tau as a central driver of disease progression [4, 5]. Yet, the mechanisms governing tau processing, trafficking, and intercellular propagation remain incompletely understood, which substantially limits the development of effective disease-modifying therapies [6, 7].

Extracellular vesicles (EVs) have emerged as an important mediator of intercellular communication in the central nervous system (CNS) and are increasingly implicated in the propagation of pathological proteins, including tau [8, 9]. EV biogenesis is tightly regulated by the endosomal system, particularly multivesicular bodies (MVBs), which control the formation and release of intraluminal vesicles [10, 11]. Dysfunctional MVBs promote AD pathological protein spread likely through at least two mechanisms: (1) impaired lysosomal fusion leading to aberrant EV-mediated secretion of MVBs-generated Aβ_42_ [12]; and (2) defective autophagosome fusion that causes accumulation of tau selectively into intraluminal vesicles for trans-neuronal transmission [13]. Furthermore, it has been demonstrated that the MVB-derived EVs carrying damaged DNA exacerbate neuroinflammation [14]. Despite these advances, the molecular machinery that governs the EV-mediated tau secretion remains incompletely understood.

Recent biomarker studies have identified phosphorylated tau species, particularly p-tau217, as sensitive indicators of AD pathology in plasma and cerebrospinal fluid (CSF) [15–17]. Beyond the diagnostic utility, these observations also provide important biological insights. Notably, while free-form p-tau217 levels are elevated in AD plasma, our previous study observed a paradoxical reduction in the proportion of p-tau217⁺ EVs [18].

Emerging evidence indicates that truncated tau is enriched in the endosome-lysosome-associated EVs and can be actively secreted, highlighting EV-mediated tau trafficking as a regulated biological process [19]. Consistently, pharmacological enhancement of tau efflux, such as with methazolamide, has been shown to improve cognitive function in mice [20]. These findings collectively indicate that tau secretion is dynamically controlled and functionally relevant to AD pathogenesis. Therefore, the observed reduction in p-tau217⁺ EVs in AD suggests disease-associated alterations in EV biogenesis or cargo loading, pointing to a previously unrecognized regulatory mechanism.

In this study, we integrated mass spectrometry-based proteomic analysis of p-tau217^+^ EVs captured from the CSF of AD patients and healthy controls (HCs) with our recent spatial transcriptomic analysis [21] to investigate the molecular mechanisms underlying impaired p-tau217^+^ EV secretion in AD. Based on these integrated analyses, we focused on heat shock protein family A member 8 (HSPA8) as a candidate regulator of MVB trafficking and SNARE complex assembly. Using Aβ_42_-treated cellular models and AD model mice, we investigated whether HSPA8 regulates p-tau217^+^ EV biogenesis and release by modulating the interaction between Aβ and SNAP29. We further evaluated the effects of genetic HSPA8 silencing and a rationally designed interfering peptide targeting this regulatory pathway. This study aimed to elucidate the mechanisms underlying defective EV-mediated p-tau217 secretion and to evaluate the therapeutic potential of targeting HSPA8-mediated regulation in AD.

## Methods

### Human subject enrollment and clinical sample collection

All experiments related to human samples were approved by the Human Ethics Committee of the School of Medicine, Zhejiang University (approval no. 2021-400). Clinical samples were obtained from the First Affiliated Hospital, Zhejiang University School of Medicine. CSF samples were collected from 21 AD patients and 22 HCs, and plasma samples were collected from 30 AD patients and 30 HCs. Extensive clinical and CSF information was collected alongside the samples (listed in Table S1). All participants underwent clinical evaluation as previously described [22, 23]. AD patients exhibited CSF biomarker profiles consistent with AD based on established cutoffs [18, 24–26]. Sample collection and processing were conducted according to previously described protocols [27].

### Cell culture and blood–brain barrier (BBB) establishment

The SH-SY5Y (RRID: CVCL_0019) cell line was obtained from the China Center for Type Culture Collection (CCTCC, Wuhan, China). The cells were maintained in DMEM/F12 (Peiyuan Biotechnology, Cat# L310KJ, Shanghai, China) supplemented with 10% heat-inactivated fetal bovine serum (Gibco, Cat# 30044333, Grand Island, NY) and antibiotics (100 U/mL penicillin, 100 µg/mL streptomycin; Peiyuan, Cat# S110JV) at 37 °C in a humidified atmosphere containing 5% CO_2_.

For BBB establishment, astrocytes were isolated from postnatal day 1 mouse cortices, digested with 0.25% Trypsin/EDTA (Peiyuan, Cat# S310KJ), filtered through a 70 μm filter, centrifuged at 1,000× *g* for 5 min, and cultured in poly-*D*-lysine-coated flasks. After 12–14 days of incubation, microglia were removed by shaking the cultures at 200 rpm at 37 ℃ overnight, and pure astrocytes were obtained by trypsinization and subsequent subculturing.

Brain microvascular endothelial cells and pericytes were isolated from the cerebra of 6–8-week-old mice, sequentially digested with collagenase II/DNase I and collagenase/neutral protease, separated by 20% bovine serum albumin gradient centrifugation, then cultured in selective media. For endothelial cell culture, the pellet was resuspended in endothelial-selective medium (DMEM/F12 supplemented with 10% fetal bovine serum, 1× penicillin–streptomycin, 1.5 ng/mL bFGF, 100 μg/mL heparin, 5 μg/mL insulin, 5 μg/mL transferrin, 5 ng/mL sodium selenite, and 4 μg/mL puromycin) and plated onto collagen type IV (0.1 mg/mL)- and fibronectin (0.1 mg/mL)-coated dishes. Puromycin was removed from the medium after 48 h. Confluent monolayers formed within 5 days. Pericytes were cultured in low-glucose DMEM on an uncoated dish and passaged 3–5 times to obtain high-purity pericytes.

The BBB model was established using a Transwell (Corning, Cat# 3470, NY) co-culture system. Astrocytes (4 × 10^4^ cells/cm^2^) were seeded into the lower chamber. Pericytes (4 × 10^4^ cells/cm^2^) were plated on the underside of the Transwell insert membrane by inverting the insert. After attachment, endothelial cells (4 × 10^5^ cells/cm^2^) were seeded on the upper side of the insert to form a confluent monolayer. Then, the cultures were maintained in BBB induction medium (DMEM/F12 containing 10% fetal bovine serum, 1× penicillin-streptomycin, 1.5 ng/mL bFGF, 100 μg/mL heparin, 8 μg/mL insulin/transferrin, 10 ng/mL sodium selenite, 550 nM hydrocortisone, 1 μM retinoic acid, 312.4 μM 8-CPT-cAMP, 17.5 μM PDE4 inhibitor RO 20-1724, and 50 μg/mL gentamicin), with medium change every 48 h. Trans-epithelial electrical resistance (TEER) was measured in triplicate using an epithelial volt-ohmmeter (World Precision Instruments, Sarasota, FL) and mean values were recorded.

### Cell viability assay

Cell viability was assessed using a CCK-8 assay kit (Abcam, Cat# ab228554, Cambridge, UK) in accordance with the manufacturer’s instructions. Cells were plated in 96-well plates, and absorbance at 450 nm was measured using a microplate reader (Molecular Devices, San Jose, CA).

### Lactate dehydrogenase (LDH) assay

Cell cytotoxicity was evaluated by measuring LDH release in the culture supernatant using a commercial kit (Beyotime, Cat# C0017, Shanghai, China) according to the manufacturer’s protocol. Absorbance at 490 nm was measured with a microplate reader (Molecular Devices).

### Animals

All animal procedures were approved by the Ethics Committee of the First Affiliated Hospital, Zhejiang University School of Medicine (approval no. 2021-667), and carried out following the ARRIVE guidelines to minimize discomfort. 5× FAD mice (JAX Stock No. 008730) were purchased from The Jackson Laboratory (Bar Harbor, ME). C57BL/6 mice (Stock No. SM-001) and APP/PS1 mice (Stock No. NMX-TG-192043) were obtained from Shanghai Model Organisms (Shanghai, China) and housed at 22 ± 2 °C with a 12-h light/dark cycle and free access to food and water.

### EV isolation from cell culture supernatant and mouse plasma

The culture medium was collected and centrifuged at 2000× *g* for 20 min at 4 °C, followed by ultracentrifugation at 100,000× *g* for 2 h at 4 °C (Beckman Coulter Optima XPN-100, SW41 Ti rotor). The pellet was resuspended in 0.22 μm-filtered PBS (pH 7.4) and ultracentrifuged again under the same conditions. The final EV-enriched pellet was resuspended in 100 μL PBS and stored at − 80 °C.

Mouse plasma samples were rapidly thawed at 37 °C and centrifuged at 2000× *g* for 15 min at 4 °C to obtain platelet-free plasma. The resulting supernatant was then centrifuged at 12,000× *g* for 30 min at 4 °C to remove cellular debris, and the final supernatant was collected. Then, 10 μL of clarified plasma samples were diluted with 70 μL of PBS and 20 μL of 40% PEG8000 (Sigma-Aldrich, Cat# 89510) following our previously established method [21].

### Analysis of EVs by CytoFLEX (VSSC mode)

EVs from cell culture supernatant or mouse plasma were measured as previously described [18, 21]. EVs (10 μL) were blocked with an equal volume of 2% BSA and incubated overnight at 4 °C with 0.05 μg primary antibodies against p-tau217 (Thermo, Cat# 44-744), p-tau181 (Thermo, Cat# MN1050), p-tau231 (Thermo, Cat# MN1040), p-tau396 (Thermo, Cat# 35-5300), and total tau (t-tau, Thermo, Cat# MA5-12808). Antibodies were fluorescently labeled with Zenon™ Alexa Fluor™ 647 labeling kits (Invitrogen, Carlsbad, CA) according to the manufacturer’s instructions, using the Rabbit IgG Labeling Kit (Cat# Z25308), Mouse IgG_1_ Labeling Kit (Cat# Z25008), or Mouse IgG_2b_ Labeling Kit (Cat# Z25208) as appropriate for each antibody isotype. Then, PBS (62 μL) and lipid probe (5 μL) were added (final volume 100 μL, probe 500 nM), followed by 1 h incubation and fixation with 100 μL 4% paraformaldehyde for 20 min. Samples were analyzed using CytoFLEX VSSC mode [21, 28], and 50,000 particles were collected under the lipid probe gate. Gates were established using a standardized calibration strategy. Silica nanosphere reference beads of defined diameters (80 nm, 100 nm, 150 nm, 200 nm, and 400 nm) were used to calibrate side scatter intensity and define the detectable particle size range of the instrument. Fluorescence-positive events were defined by setting thresholds above background fluorescence determined using immunoglobulin isotype controls and dye-only blank controls [18]. Based on these criteria, gates were applied to distinguish particle populations (upper-right quadrant of the flow cytometry plot) that exceeded the signals of unlabeled particles and were thus recognized as positive events by the nanoflow cytometer.

### Intravesicular and surface p-tau217 analysis of human plasma EVs

EVs of human plasma were permeabilized with 1% Triton X-100 in PBS and incubated for 30 min at 25 °C [29], followed by three washes with PBS. The permeabilized EVs were then incubated with a fluorophore-conjugated p-tau217 antibody for labeling as described above, while intact EVs were labeled in parallel as controls. Samples were subsequently analyzed using CytoFLEX in VSSC mode.

### Immunocapture of p-tau217^+^ EVs

EVs were isolated from 5 mL of pooled human CSF by ultracentrifugation. Thawed CSF samples were sequentially centrifuged at 2000× *g* for 15 min and 12,000× *g* for 30 min at 4 °C to remove debris. The supernatant was diluted 1:10 with 0.22 μm-filtered PBS (pH 7.4) and ultracentrifuged at 100,000× *g* for 1 h at 4 °C (Beckman Coulter Optima MAX-XP, TLA-55 rotor, Brea, CA). The pellet was washed with 0.22 μm-filtered PBS (pH 7.4) once, ultracentrifuged under the same conditions, resuspended in 100 μL PBS, and stored at − 80 °C.

The p-tau217^+^ EVs were immunocaptured from human CSF-derived EVs using an anti-p-tau217 antibody (Invitrogen, Cat# 44744) conjugated to magnetic beads (Dynabeads™ Antibody Coupling Kit, Invitrogen, Cat# 14311D) according to the manufacturer’s instructions. Briefly, 10 μg of anti-p-tau217 antibody was biotinylated overnight at 4 °C and conjugated to streptavidin beads. The EVs (500 μL) were incubated with magnetic beads (diluted 1:1.5 with PBS) for 24 h at 4 °C with gentle rotation. After PBS washes, EVs were eluted with 0.1 M glycine (pH 2.8) and neutralized with Tris buffer (pH 7.4).

### Cryo-electron microscopy (Cryo-EM)

EV-enriched fractions from plasma or CSF (5 μL) were loaded onto perforated carbon-coated EM grids and incubated briefly to allow particle attachment. The grids were subsequently vitrified by rapid immersion in liquid ethane using a Vitrobot device (Thermo Fisher Scientific, Waltham, MA). Cryo-EM imaging was performed with a Talos F200C transmission electron microscope (Thermo Fisher) at an accelerating voltage of 200 kV.

### Nanoparticle tracking analysis (NTA)

The size distribution and concentration of EVs were measured using a NanoSight NS300 system. EV samples (10 μL) were diluted in 0.22 μm filtered PBS to a final volume of 1 mL prior to measurement. Particle movement driven by Brownian motion and the corresponding light scattering signals were recorded by the instrument, and three 60-s videos were collected for each sample. Data were processed with NTA software (version 3.2, NanoSight, Amesbury, UK) using the same threshold parameters for all groups.

### Mass spectrometry

CSF-derived p-tau217^+^ EVs were lysed in 8 M urea buffer (pH 8.5, Sigma, Cat# 33247) with 1% protease inhibitors, sonicated and centrifuged at 12,000× *g* for 10 min at 4 °C. Protein was quantified via BCA assay (Invitrogen, Cat# 23227). Aliquots (100 μg) were reduced (5 mM DTT, 65 °C, 30 min), alkylated (11 mM iodoacetamide, 15 min), then buffer-exchanged to 0.1 M NH_4_HCO_3_ using 10 kDa filters. Trypsin digestion (1:50 *w*/*w*, 37 °C, > 12 h) was terminated by trifluoroacetic acid acidification and lyophilization. Peptides were desalted (C18 stage tips), reconstituted in 0.1% TFA, then in bRP buffer A (10 mM NH_4_HCO_3_/pH 10, 5% ACN) for Orbitrap Exploris 480 analysis (1 μg load).

Mass spectrometry data were processed using Proteome Discoverer 2.5 (SEQUEST, SwissProt Human) with tryptic digestion, ≤ 2 missed cleavages, ≥ 6-aa peptides, and ≤ 5 modifications. Mass tolerances: 10 ppm (precursors) and 0.02 Da (fragments). Modifications: fixed modification, cysteine carbamidomethylation; variable modifications, methionine oxidation, and N-terminal acetylation/met-loss. False discovery rate was set at 1% for peptide-spectrum matches and proteins.

### Quantitative real-time PCR

Cells were washed with PBS, lysed in 1 mL Trizol (Invitrogen, Cat# 15596018CN), and incubated on ice for 30 min. After adding 200 μL chloroform and vortexing, samples were incubated on ice (10 min) and centrifuged at 12,000× *g* for 10 min at 4 °C. The aqueous phase was mixed with isopropanol, incubated on ice (10 min), and centrifuged again. The RNA pellet was washed twice with 75% ethanol, air-dried, dissolved in nuclease-free water, and quantified by NanoDrop.

Genomic DNA was removed prior to cDNA synthesis using the PrimeScript RT Reagent Kit (Takara Bio, Cat# RR047A, Kusatsu, Japan). qPCR was performed with Green® Premix Ex Taq™ (Takara Bio, Cat# RR420A) on a BIO-RAD CFX384 system. Primers are listed in Table S2.

### Western blotting and co-immunoprecipitation (Co-IP)

Protein samples were extracted using RIPA buffer and centrifuged at 14,000× *g* for 10 min at 4 °C. The supernatants were collected, and protein concentrations were determined using a BCA assay (Invitrogen, Cat# 23227). Equal amounts of protein were separated by SDS-PAGE and transferred onto PVDF membranes. After blocking with 5% skim milk, the membranes were incubated with primary antibodies (Table S3) at 4 °C overnight. All primary antibodies were diluted in universal antibody diluent (NCM Biotech, WB100D, Suzhou, China). After washing with TBST, the membranes were incubated with IRDye 800CW-conjugated secondary antibodies (1:10,000; LI-COR Biosciences, Cat#926-32210 or 926-32213, Lincoln, NE) for 2 h at room temperature. Protein bands were visualized using a ChemiDoc™ imaging system (BIO-RAD Laboratories, Hercules, CA).

Co-IP was performed using the Pierce™ Classic Magnetic IP/Co-IP Kit (Thermo Fisher Scientific, Cat# 88804) according to the manufacturer’s protocol. For each reaction, 1000 μg of protein lysate was incubated with 2 μg of anti-HSPA8 antibody (Abcam, Cat# ab51052) in 500 μL IP lysis/wash buffer overnight at 4 °C with rotation. Pierce™ Protein A/G Magnetic Beads (25 μL; Thermo Fisher Scientific, Cat# 88804) were washed three times with IP buffer before adding the antigen–antibody complexes. After 2-h incubation at room temperature with rotation, beads were collected magnetically and washed three times with IP buffer, followed by ultrapure water. Bound proteins were eluted with 100 μL elution buffer for 15 min at room temperature, neutralized with 10 μL neutralization buffer, and analyzed by Western blotting.

### ELISA

CSF and plasma levels of p-tau217 were quantified using the PathScan® Phospho-Tau (Thr217) ELISA Kit (Cell Signaling Technology, Cat# 87749C, Danvers, MA) following the manufacturer’s protocol. Samples were diluted to fall within the standard curve, mixed 1:1 with detection antibody (25 µL each), and incubated for 1 h at 400 rpm. After four washes, chemiluminescence was read at 425 nm within 10 min of substrate addition.

### Immunofluorescence staining

Immunofluorescence staining was performed on both frozen brain sections (15 µm) and cultured cells, which were fixed with 4% paraformaldehyde for 20 min. After PBS washing and permeabilization with 0.3% Triton X-100, samples were blocked with 3% BSA solution for 30 min at room temperature and incubated overnight at 4 °C with primary antibodies (Table S3). After washing, samples were incubated with fluorophore-conjugated secondary antibodies (1:500, Invitrogen, Cat# A32723, A11008, A10520, A21236, and A21449) for 2 h, counterstained with DAPI (Vector Laboratories, Cat# H-1400, Newark, CA), and imaged by STELLARIS 8 confocal microscopy (Leica Microsystems, Mannheim, Germany).

### LysoTracker red staining

SH-SY5Y cells were seeded onto confocal imaging dishes and incubated with 50 nM LysoTracker Red DND-99 (Thermo Fisher, Cat# L7528) at 37 °C for 30 min to label lysosomes. After staining, the cells were fixed at room temperature with 4% paraformaldehyde, followed by immunostaining with antibodies against p-tau217 and CD9, as well as nuclear counterstaining with DAPI. Imaging was performed using a Leica STELLARIS 8 confocal microscope.

### Stereotaxic injection in the hippocampus

Anesthetized WT and APP/PS1 mice were positioned in a stereotaxic frame (RWD Life Science, Shenzhen, China) for unilateral hippocampal injections (1.7 mm posterior and 1.4 mm lateral from bregma, 2.0 mm depth). For viral vector experiments, 0.1 μL of either pAV-hsyn-GFP-miR30-shRNA or its control vector (Vigene Biosciences, Rockville, MD) was injected at a rate of 0.2 μL/min using a Hamilton syringe. In peptide experiments, 0.5 μL of either SNAP29 25-mer peptide (Sequence: QEAKYQASHPNLRKLDDTDPVPRGA) or scramble peptide was infused at the same rate. The needle was held in place for 10 min post-injection. Behavioral assessments were conducted 1 month after injection (at 6 months of age) of pAV-hsyn-GFP-miR30-shRNA or control vector, or tested 1 week after 25-mer peptide or scramble peptide injection (at 8 months).

### Behavioral assessments

Open Field Test: Mice freely explored a 60 × 60 cm^2^ arena for 5 min, and the total distance was analyzed using ANY-maze Video Tracking System (Stoelting Co., Wood Dale, IL).

Novel Object Recognition: Mice were habituated (Day 1) in a 40 × 40 cm^2^ arena for 10 min, trained with two identical objects (Day 2), and tested with one novel object (Day 3) for 10 min. The exploration times were recorded.

Y-Maze Test: Mice explored a Y-shaped maze (three arms, 120° angles) for 5 min. Spontaneous alternations were automatically quantified using an ANY-maze Video Tracking System.

Morris Water Maze: Mice were trained in a 120-cm-diameter pool (20–22 °C, opaque water) with a submerged platform (1 cm depth) over six days (two trials/day, 60 s/trial). Escape latency and path efficiency were recorded. If mice failed to locate the platform within 60 s, they were guided to it and allowed to remain there for 10 s. A 60-s probe trial (platform removed) was performed on day 6 to assess spatial memory by measuring target quadrant crossings. Swimming trajectories were tracked using EthoVision XT 16 (Noldus).

### Molecular docking

Models of HSPA8 substrate-binding domain bound to SNAP29 were generated using the online AlphaFold Server, available at https://alphafoldserver.com/.

### Cell transfection

Flag-hTau T217D or Flag-hTau T217A plasmids (Shanghai Genechem Co., Ltd) were transfected into SH-SY5Y cells with Lipofectamine 3000 Transfection Reagent (Invitrogen, Cat# L3000150).

3× Flag-tagged HSPA8 (Human) cDNA was cloned into KV233 plasmids. HA tag, HA-SNAP29 Full-length (Human), HA-SNAP29 156-180-EGFP or HA-SNAP29 Δ156-180 cDNA was cloned into GV658 plasmids, separately. All vectors were purchased from Shanghai Genechem Co., Ltd and delivered into HEK293T cells with Lipo8000™ Transfection Reagent (Beyotime Biotechnology, Cat# C0533).

The HA-tagged truncated SNAP29 and 3× Flag-tagged HSPA8 cDNAs were co-transfected into HEK293T cells. After 24 h of transfection, the whole-cell lysates were immunoprecipitated with anti-Flag beads (Thermo Fisher Scientific, Cat# A36797), and immunoblotted with the HA antibody (Thermo Fisher Scientific, Cat# 26183).

### Statistical analysis

All experimental measurements are presented as Means ± standard error of the mean (SEM). All data were assessed for normality using the Shapiro–Wilk test within each group. For data with a normal distribution, two-tailed Student’s *t*-test was used for comparisons between two groups, and one-way ANOVA followed by the Tukey–Kramer post-hoc test was applied for comparisons among three or more groups. For data that did not follow a normal distribution, the non-parametric Mann–Whitney U test was used for two-group comparisons, and the Kruskal–Wallis test was used for comparisons across multiple groups. Graphical representations and statistical analyses were performed using GraphPad Prism 9.0 (GraphPad Prism Software, La Jolla, CA), with statistical significance defined as *P* < 0.05.

## Results

### Periphery transport is not responsible for reduced peripheral blood p-tau217^+^ EVs

While elevated p-tau217 levels in AD brain and peripheral blood have been reported [30], we previously identified a reduced proportion of p-tau217^+^ EVs in AD plasma [18]. To further explore the underlying mechanisms, we measured the levels of free-form p-tau217 in the CSF and plasma of AD patients and HC subjects in samples from the same source as the aforementioned study [18]. The results showed significantly higher free-form p-tau217 levels in the CSF of AD patients compared to HC (Fig. S1a). In plasma, AD patients also exhibited significantly higher levels of free-form p-tau217 than HC (Fig. S1b). These findings, consistent with previous studies, confirmed increased levels of free-form p-tau217 in both CSF and plasma in AD.

To investigate whether the transport capacity of p-tau217^+^ EVs from the CNS to peripheral blood is impaired in AD, we established an in vitro three-cell BBB model based on our previous study [31]. Immunofluorescence staining showed that the primary cultured astrocytes, brain microvascular endothelial cells, and pericytes highly expressed glial fibrillary acidic protein (GFAP), von Willebrand factor (vWF), and neuron-glial antigen 2 (NG2), respectively (Fig. S2a). The BBB model exhibited robust barrier integrity, with TEER gradually increasing from a baseline of approximately 21 Ω cm^2^ (cell-free chamber) to a plateau of 405 Ω cm^2^ by day 5 (Fig. S2b). Human peripheral blood plasma EVs were labeled with fluorophore-conjugated p-tau217 antibody. Equal amounts of labeled EVs, as previously characterized in our prior study [18], were added to both the intact and the 5 μM Aβ_42_-treated BBB models. Nanoflow cytometry results revealed a significantly higher proportion of EVs crossing the Aβ_42_-treated BBB model (Fig. S2c, d), suggesting that the reduced proportion of p-tau217^+^ EVs in the peripheral blood in AD is unlikely due to impaired transport from the CNS.

### Reduced synthesis/secretion of p-tau217^+^ EVs

**Fig. 1:**
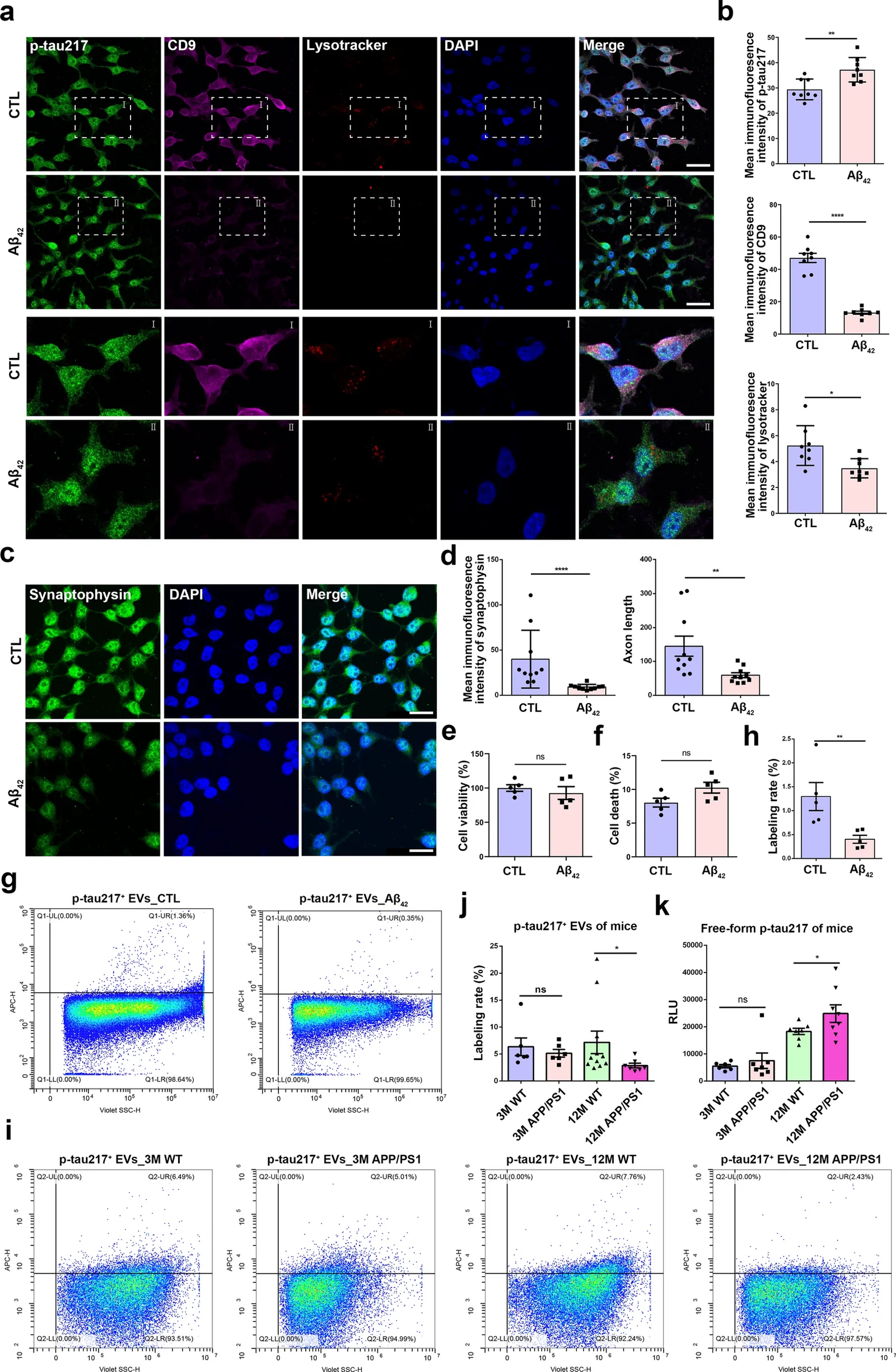
The level of p-tau217 and p-tau217^**+**^ EVs in Aβ_42_-treated cells or APP/PS1 mice. **a** Representative immunocytochemical images for p-tau217 (green), CD9 (purple), and Lysotracker (red) in SH-SY5Y cells with or without Aβ_42_ treatment. Nuclei were stained with DAPI. Scale bars: 50 µm. **b** Quantification of the fluorescence intensity for p-tau217, CD9 and lysotracker in SH-SY5Y cells. *n* = 8 in each group. **c** Immunocytochemical images for Synaptophysin in SH-SY5Y cells with or without Aβ_42_ treatment. Scale bars: 50 µm. **d** Quantification of the immunofluorescence intensity for Synaptophysin and axon length. *n* = 10 in each group. **e** Cell viability of SH-SY5Y cells treated with or without Aβ_42_ was detected by CCK-8 assay. *n* = 5 in each group. **f** Cell death of SH-SY5Y cells treated with or without Aβ_42_ was assessed by LDH release assay. *n* = 5 in each group. **g** Nanoflow cytometry analysis showing the percentage of p-tau217^+^ EVs in SH-SY5Y cells. **h** Quantification of the percentage of p-tau217^+^ EVs in the supernatant of SH-SY5Y cells. *n* = 5 in each group. **i** Nanoflow cytometry analysis showing the percentage of p-tau217^+^ EVs in the mouse plasma. **j** Quantification of the percentage of p-tau217^+^ EVs in plasma. *n* = 6 for 3-months WT and 3-months AD mice, 11 for 12-months WT mice, and 7 for 12-months AD mice. **k** The level of free-form p-tau217 in plasma. *n* = 6 for 3-months WT mice, 7 for 3-months AD mice and 12-months WT mice, and 8 for 12-months AD mice. All values are means ± SEM. ^*^P < 0.05, ^**^P ﻿< 0.01, ^****^P﻿ < 0.0001. ns, no significance

After excluding impaired transport capacity to the periphery, we subsequently evaluated the degradation of p-tau217^+^ EVs under the Aβ_42_ treatment condition. SH-SY5Y cells treated with 5 μM Aβ_42_ showed significantly higher fluorescence intensity of p-tau217 compared to the control group (Fig. 1a, b), indicating intracellular accumulation of p-tau217 in AD. Additionally, the fluorescence intensity of CD9-labeled EVs in the Aβ_42_-treated group was significantly lower (Fig. 1a, b), indicating impaired EV synthesis under Aβ_42_ stimulation. The fluorescence intensity of active lysosomes, as labeled by Lysotracker, was significantly reduced in the Aβ_42_-treated group (Fig. 1a, b), suggesting a marked decrease in lysosomal degradation capacity in AD. These findings suggest that while lysosomal degradation is compromised, p-tau217^+^ EV synthesis/secretion is more severely affected, implicating impaired production of p-tau217^+^ EVs as the primary cause of reduced peripheral p-tau217^+^ EVs in AD.

Furthermore, the fluorescence intensity of synaptophysin, a key protein reflecting synaptic function involved in synapse formation, transmission, and plasticity, was significantly reduced in the Aβ_42_-treated group, and axon length was notably shortened (Fig. 1c, d), suggesting synaptic damage and impaired synaptic function in Aβ_42_-treated neurons. However, CCK-8 and LDH assays revealed no significant changes in cell viability or cytotoxicity following Aβ_42_ treatment **(**Fig. 1e, f**)**. EVs extracted from culture supernatants (characterized in Fig. S3a-c) revealed a marked decrease in p-tau217^+^ EVs in the Aβ_42_-treated group (Fig. 1g, h), indicating a reduced capacity of neurons to synthesize and secrete p-tau217^+^ EVs under AD conditions, rather than reflecting Aβ-induced cell death.

To further determine whether this phenomenon extends to other tau phospho-epitopes, we analyzed additional phosphorylation sites in the Aβ_42_-treated SH-SY5Y cells. Consistent with enhanced tau phosphorylation, immunostaining showed that intracellular levels of p-tau181, p-tau231, and p-tau396 were all increased (Fig. S4a-c). Nanoflow cytometry analysis revealed that, like EV-associated p-tau217, the EV-associated p-tau181⁺ and p-tau396⁺ populations were significantly reduced, whereas the p-tau231⁺ EVs showed no significant change (Fig. S4e-g). Importantly, the t-tau levels remained unchanged both intracellularly and in EVs following Aβ_42_ treatment, indicating that the observed alterations were not due to changes in overall tau expression (Fig. S4d, h). These findings further support that Aβ_42_ primarily impairs the selective packaging and secretion of phosphorylated tau species via EVs, rather than affecting total tau production.

These findings were further validated in the peripheral blood of WT and APP/PS1 mice using plasma EVs (characterized in Fig. S3d-f). Nanoflow cytometry analysis showed no significant difference in the proportion of plasma p-tau217⁺ EVs between 3-month-old WT (3 M WT) and APP/PS1 (3 M AD) mice, while the 12 M AD mice exhibited a significant reduction compared to 12 M WT mice (Fig. 1i, j). Meanwhile, the levels of plasma free-form p-tau217 were comparable between 3 M WT and 3 M AD mice but significantly elevated in 12 M AD mice (Fig. 1k), consistent with age-dependent AD pathology.

These in vivo and in vitro findings indicate that the impaired neuronal synthesis and secretion of p-tau217⁺ EVs contribute to reduced EV-mediated transport and neuronal accumulation of p-tau217.

### HSPA8 modulates the SNARE complex and secretion of p-tau217^+^ EVs

**Fig. 2:**
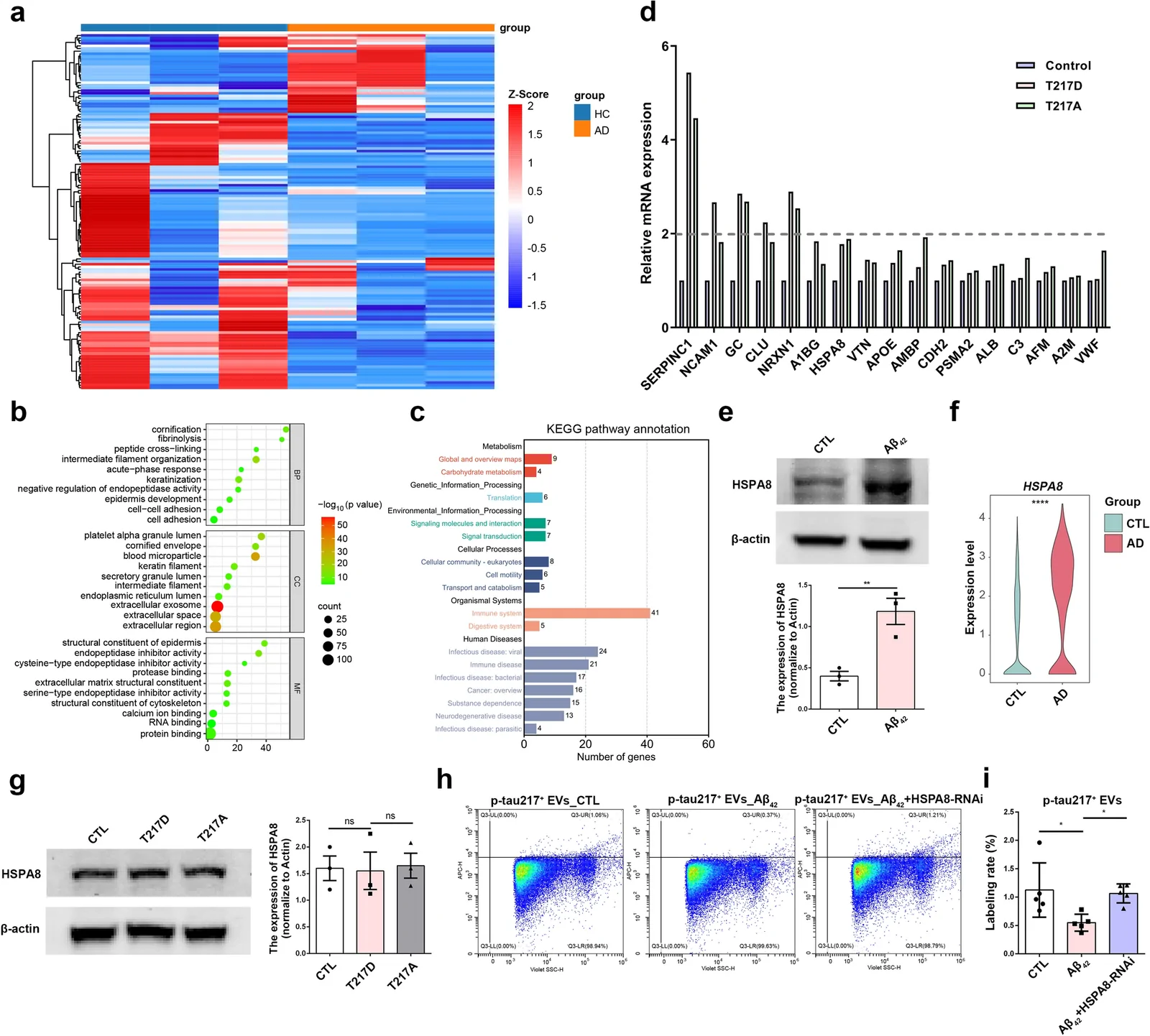
HSPA8 is an upstream signaling molecule that regulates p-tau217-associated EVs. **a** Clustering analysis of DEGs in CSF-derived EVs from HC and AD patients. The heatmap displays the DEGs, with blue indicating downregulation and red indicating upregulation. *n* = 3 for HC and AD, each sample consisting of equal volumes of CSF pooled from 5 HC individuals or 5 AD patients. **b** GO enrichment analysis of DEGs in p-tau217^+^ EVs from human CSF. **c** KEGG pathway analysis of DEGs in p-tau217^+^ EVs from human CSF. **d** mRNA expression of Top 15 hub genes in SH-SY5Y cells under simulated tau phosphorylation/dephosphorylation at Thr217. The histogram shows the average values of three replicates. **e** Protein expression of HSPA8 in SH-SY5Y cells with or without 5 μM Aβ_42_. *n* = 3 in each group. **f** Expression of *HSPA8* gene in human hippocampus in CTL and AD. *n* = 8 in each group. **g** Protein expression of HSPA8 in tau phosphorylation/dephosphorylation at Thr217. *n* = 3 in each group. **h** Nanoflow cytometry analysis showing the impact of HSPA8 knockdown on p-tau217^+^ EVs in SH-SY5Y cells with 5 μM Aβ_42_. **i** Quantification of the percentage of p-tau217^+^ EVs in the supernatant of SH-SY5Y cells. *n* = 5 in each group. All data are shown as means ± SEM. ^*^P﻿ < 0.05, ^**^P﻿﻿ < 0.01, ^****^P﻿ < 0.0001. ns, no significance

To further identify key pathways and targets involved in p-tau217⁺ EV synthesis and secretion, we immunocaptured the p-tau217^+^ EVs from CSF of AD patients and HC subjects for mass spectrometry analysis. The captured EVs were validated by cryo-EM and NTA (Fig. S3g-i), after verifying that p-tau217 is present both on the EV surface and within the vesicular lumen (Fig. S3j). The results revealed that p-tau217^+^ EVs exhibited 44 significantly upregulated proteins and 81 significantly downregulated proteins in AD compared to HC (Fig. 2a). We then performed enrichment analysis of the differentially expressed genes (DEGs) using GO and KEGG databases. GO functional enrichment analysis revealed that the DEGs were enriched in components such as extracellular exosomes, blood microparticles, and secretory granule lumen (Fig. 2b). KEGG pathway analysis showed that the DEGs were involved in pathways related to neurodegenerative diseases and the immune system (Fig. 2c). To further explore the core genes in the differential protein interaction network, we performed protein–protein interaction (PPI) analysis, followed by network analysis using Cytoscape’s cytoHubba plugin. We identified the top 15 nodes based on node scores, which were identified as the Top 15 Hub genes (Table S4).

To explore the regulatory relationship between hub genes and p-tau217^+^ EV secretion, we simulated tau phosphorylation state at threonine 217 (T217) using T217D (phospho-mimetic) and T217A (dephospho-mimetic) mutants. qPCR analysis revealed that several genes from the Top 15 Hub list, including *SERPINC1, NCAM1, GC, CLU,* and *NRXN1*, were significantly altered by tau T217 phosphorylation, suggesting they might be downstream of p-tau217-related EVs (Fig. 2d). In contrast, genes including *A1BG, HSPA8, VTN, APOE, AMBP, CDH2, PSMA2, ALB, C3, AFM, A2M,* and *VWF* remained unchanged, indicating they may function as upstream regulators (Fig. 2d).

Among these genes, *HSPA8* has been reported to regulate SNARE complex function, which mediates vesicle synthesis and secretion [32]. Therefore, we hypothesized that HSPA8 may act as an upstream regulator of p-tau217⁺ EV synthesis and secretion. Western blotting showed that the HSPA8 protein level was significantly increased in Aβ_42_-treated cells (Fig. 2e). Using the human hippocampal spatial transcriptomic database from the CNGB Nucleotide Sequence Archive (accession code: CNP0005077), we further found that *HSPA8* gene expression was significantly elevated in AD hippocampus compared to those in control individuals (Fig. 2f). However, HSPA8 protein level was unaffected by tau T217 phosphorylation status (Fig. 2g), suggesting that HSPA8 is not a downstream target of p-tau217. To further confirm its regulatory role, we constructed HSPA8 knockdown shRNAs and selected HSPA8 RNAi (#19757-1) for subsequent experiments, as it most effectively reduced HSPA8 protein level (Fig. S5). Nanoflow cytometry analysis showed that the proportion of p-tau217^+^ EVs secreted into the culture medium was significantly reduced in the Aβ_42_-treated group compared to the control group. HSPA8 knockdown significantly restored p-tau217⁺ EV secretion in the Aβ_42_-treated group (Fig. 2h, i), supporting its role as a negative regulator of p-tau217⁺ EV release.

### HSPA8 regulates SNAP29–Aβ interaction to affect p-tau217^+^ EV secretion

**Fig. 3:**
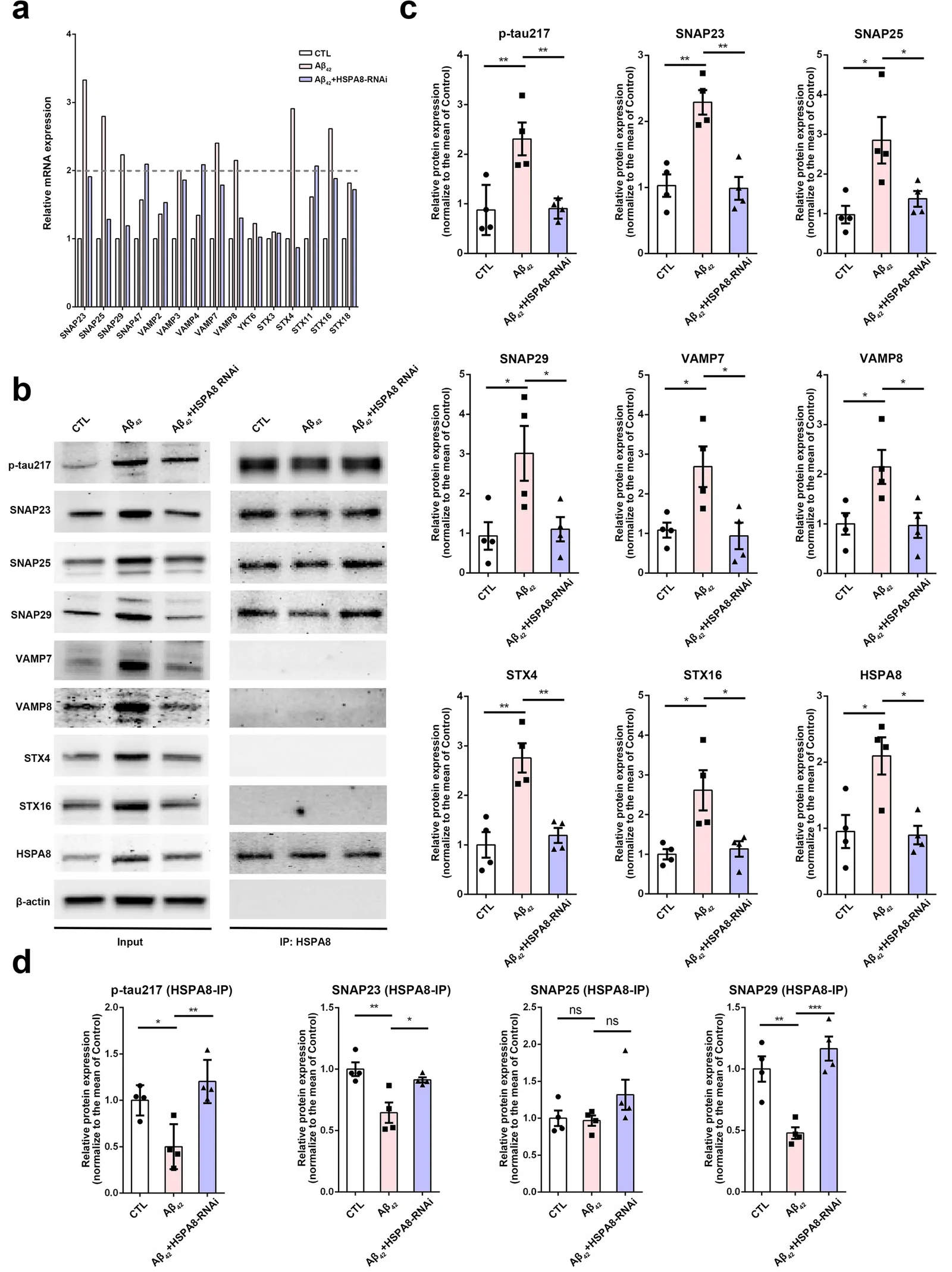
The interaction between HSPA8 and components of the SNARE complex. **a** The mRNA expressions of SNARE complex-related components. *n* = 3, with the histogram displaying the average values of three replicates. **b** Protein expressions of p-tau217, SNARE complex components (SNAP23, SNAP25, SNAP29, VAMP7, VAMP8, STX4, STX16), and HSPA8 in Input samples (left), and the interaction between HSPA8 and SNARE complex components in IP samples (right). **c** Quantification of the Western blotting results in the Input samples. **d** Quantification of the IP results showing the interaction between HSPA8 and the SNARE complex. All data are presented as means ± SEM. *n* = 4 in each group. ^*^P﻿ < 0.05, ^**^P﻿﻿ < 0.01, ^***^P﻿ ﻿< 0.001. ns, no significance

HSPA8 could affect the function of the SNARE complex, which mediates vesicle synthesis and secretion [32]. To further explore the mechanism by which HSPA8 regulates the secretion of p-tau217^+^ EVs, we investigated whether the expression levels of the SNARE complex key components were affected by HSPA8 under AD conditions. qPCR analysis revealed significant upregulation of *SNAP23, SNAP25, SNAP29, VAMP7, VAMP8, STX4,* and *STX16* in the AD model, which was partially reversed by HSPA8 knockdown (Fig. 3a).

Protein levels of p-tau217, HSPA8, and key SNARE components (SNAP23, SNAP25, SNAP29, VAMP7, VAMP8, STX4, STX16) were significantly elevated in the Aβ_42_-treated group, and all were significantly reduced following HSPA8 knockdown (Fig. 3b, c). Co-IP assays revealed significantly weakened interactions of HSPA8 with p-tau217, SNAP23, and SNAP29 in AD, which were markedly enhanced after HSPA8 knockdown (Fig. 3b, d). However, the results showed no significant changes in the interaction between HSPA8 and SNAP25 in either the Aβ_42_-treated condition or after HSPA8 knockdown (Fig. 3b, d). Additionally, no significant interactions of HSPA8 with VAMP7, VAMP8, STX4, or STX16 were detected (Fig. 3b).

**Fig. 4:**
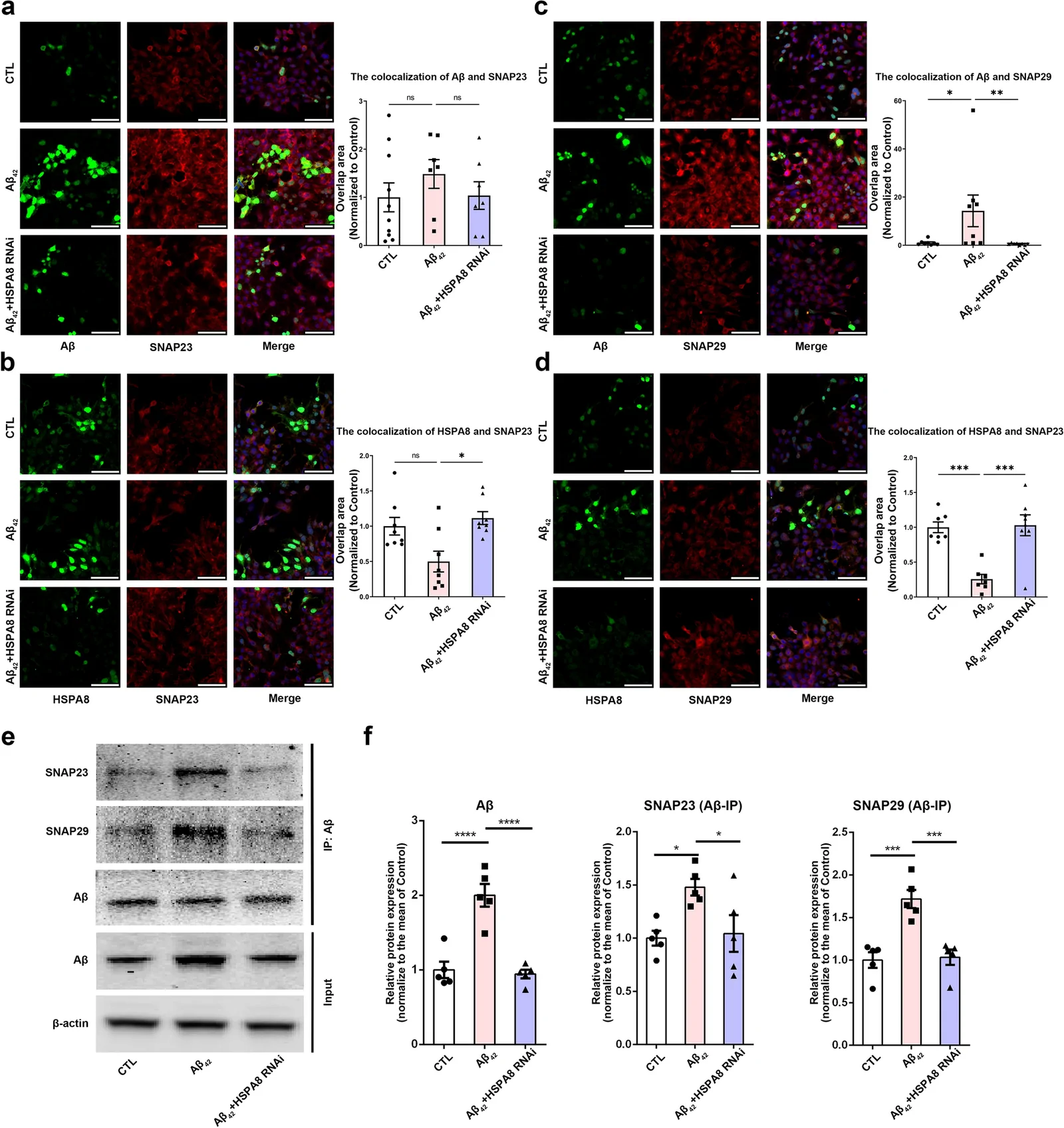
The interactions between HSPA8, SNAP23, SNAP29, and Aβ in vitro. **a** Colocalization of Aβ and SNAP23 shown by immunofluorescence (left) and quantification of the colocalization area (right). Nuclei were stained with DAPI. Scale bars: 50 µm. **b** Colocalization of HSPA8 and SNAP23 shown by immunofluorescence (left) and quantification of the colocalization area (right). Scale bars: 50 µm. **c** Colocalization of Aβ and SNAP29 shown by immunofluorescence (left) and quantification of the colocalization area (right). Scale bars: 50 µm. **d** Colocalization of HSPA8 and SNAP29 shown by immunofluorescence (left) and quantification of the colocalization area (right). Scale bars: 50 µm. **e** The interactions between SNAP23, SNAP29 and Aβ (top), and the protein expression of Aβ (bottom) in SH-SY5Y cells. **f** Quantification of Aβ expression in the Input samples (left), and statistical analysis of the IP results for the interactions between SNAP23, SNAP29, and Aβ (right). *n* = 5 in each group. All data are presented as means ± SEM. ^*^P﻿ < 0.05, ^**^P﻿﻿ < 0.01, ^***^P﻿﻿ < 0.001, ^****^P﻿﻿ < 0.0001. ns, no significance

Aβ disrupts the assembly of a functional SNARE complex by binding to SNAP proteins, thereby impairing EV secretion in AD [33]. To verify whether elevated HSPA8 in AD promotes the binding of Aβ to SNAP23 and SNAP29, we performed immunofluorescence colocalization assays. The results showed increased colocalization between Aβ and SNAP23 in AD, whereas colocalization of HSPA8 and SNAP23 exhibited a tread of decrease. HSPA8 knockdown reversed these patterns (Fig. 4a, b). On the other hand, the Aβ–SNAP29 colocalization was significantly increased, while the HSPA8–SNAP29 colocalization was significantly decreased, in Aβ_42_-treated SH-SY5Y cells. Knockdown of HSPA8 significantly reduced the Aβ–SNAP29 interaction and restored the level of HSPA8–SNAP29 colocalization (Fig. 4c, d). Co-IP results showed that the intracellular levels of Aβ in Aβ_42_-treated cells were significantly elevated, with enhanced interactions between Aβ and both SNAP23 and SNAP29. HSPA8 knockdown markedly reduced these interactions (Fig. 4e, f). In addition, we also explored the colocalization of t-tau with SNAP23 and SNAP29 as controls, but no significant changes were observed (Fig. S6).

Together, these findings demonstrate that targeted manipulation of HSPA8 in SH-SY5Y cell models directly regulates EV secretion and EV-associated p-tau217 levels, supporting a causal role of HSPA8 in EV-mediated p-tau release.

**Fig. 5:**
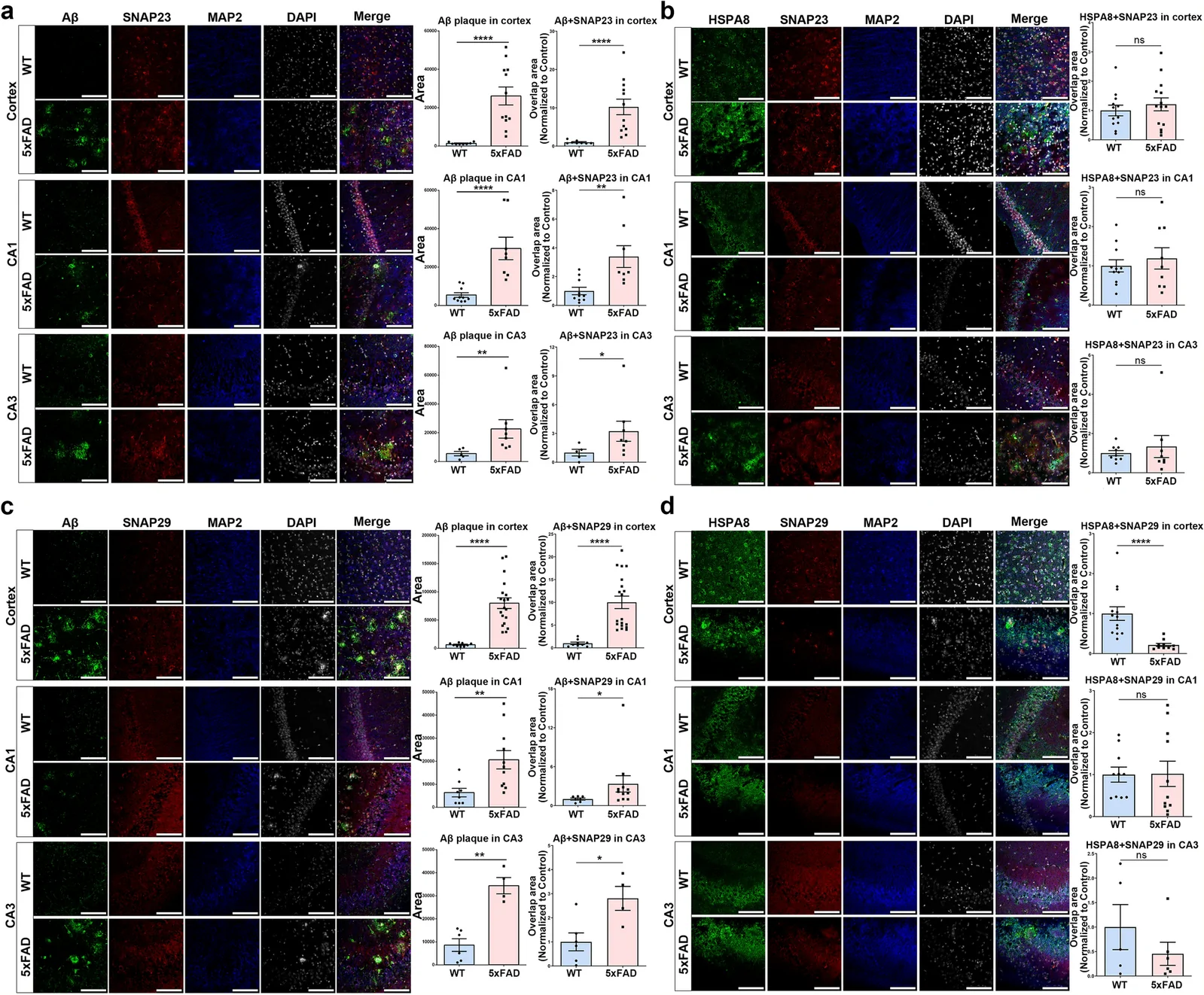
The interactions between HSPA8, SNAP23, SNAP29, and Aβ in vivo. **a** Colocalization of Aβ and SNAP23 shown by immunofluorescence and quantification of Aβ plaque and the colocalization area in cortex, CA1, and CA3. Nuclei were stained with DAPI. Scale bars: 100 µm. **b** Colocalization of HSPA8 and SNAP23 shown by immunofluorescence and quantification of the colocalization area in cortex, CA1, and CA3. Scale bars: 100 µm. **c** Colocalization of Aβ and SNAP29 shown by immunofluorescence and quantification of Aβ plaque and the colocalization area in cortex, CA1, and CA3. Scale bars: 100 µm. **d** Colocalization of HSPA8 and SNAP29 shown by immunofluorescence and quantification of the colocalization area in cortex, CA1, and CA3. Scale bars: 100 µm. *n* = 5 in each group. All data are presented as means ± SEM. ^*^P﻿ < 0.05, ^**^P﻿ < 0.01, ^****^P﻿ < 0.0001. ns, no significance

To confirm these findings in vivo, we assessed the colocalization of HSPA8 or Aβ with SNAP23 and SNAP29 in AD-related key brain regions. Aβ–SNAP23 colocalization was significantly increased in the cortex, CA1, and CA3 of 5 × FAD mice (Fig. 5a), with no significant changes in CA4 or DG (Fig. S7a). Consistently, Aβ plaque burden was significantly increased in the cortex and hippocampal subregions (Fig. 5a, Fig. S7a). In contrast, no significant differences in HSPA8–SNAP23 colocalization were observed between WT and 5 × FAD mice across the cortex and hippocampal subregions (Fig. 5b, Fig. S7b). Aβ–SNAP29 colocalization was significantly increased in the cortex, CA1, and CA3 regions of 5 × FAD mice (Fig. 5c), with an increasing trend observed in CA4 and DG (Fig. S7c). Correspondingly, the area of Aβ plaques was remarkably elevated in the cortex and hippocampus (Fig. 5c, Fig. S7c). HSPA8–SNAP29 colocalization was significantly reduced in the cortex, with decreasing trends in CA1, CA3, CA4, and DG (Fig. 5d, Fig. S7d). These results suggest that changes in the colocalization of Aβ with SNAP29, as well as HSPA8 with SNAP29, are more pronounced, indicating that HSPA8 more prominently affects AD progression by disrupting the Aβ–SNAP29 interaction.

### Inhibiting HSPA8 improves cognition and restores p-tau217^+^ EV secretion

**Fig. 6:**
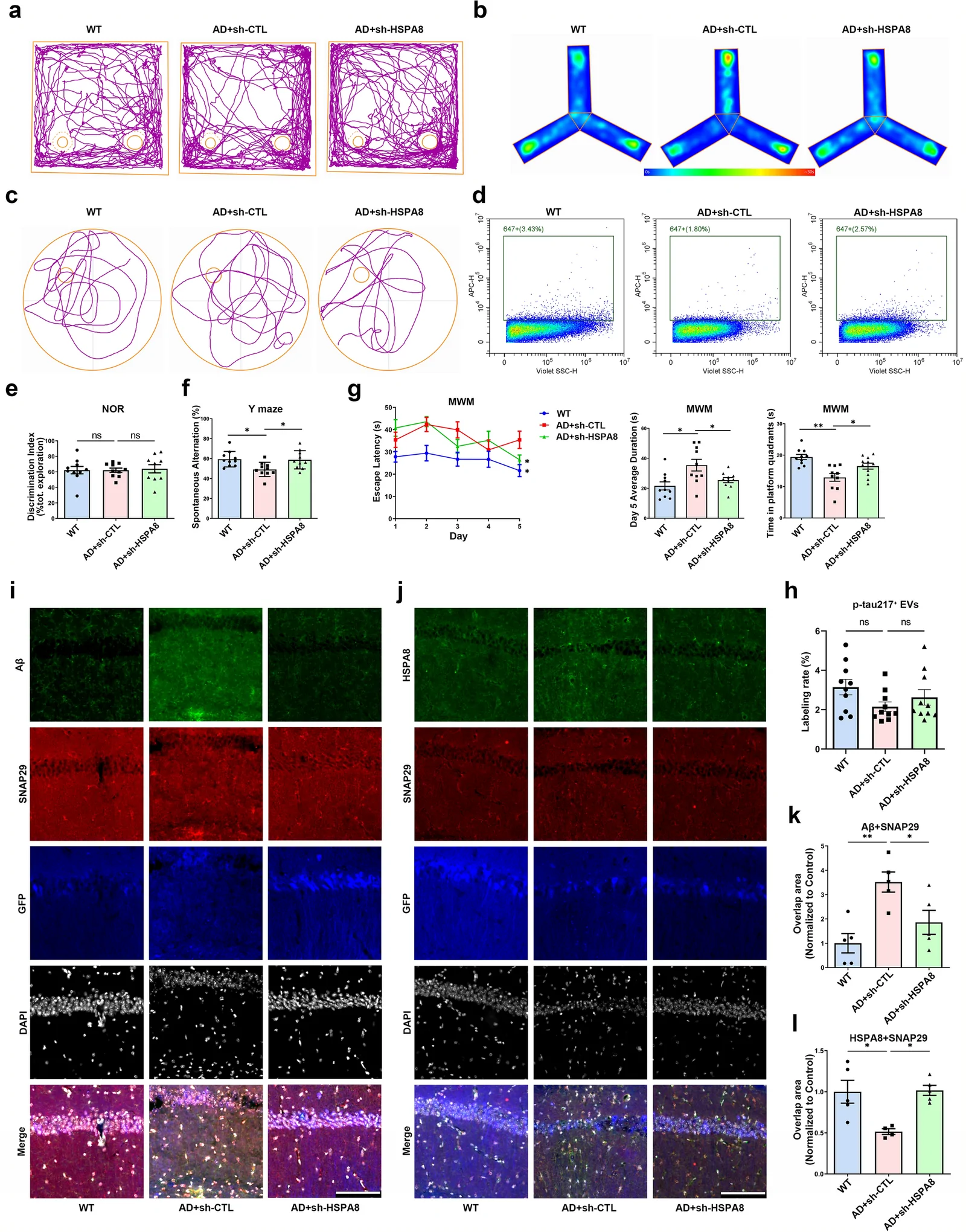
Downregulation of HSPA8 restores cognitive function and p-tau217^**+**^ EV secretion in APP/PS1 mice. **a** Representative images from the novel object recognition (NOR) test. **b** Representative trajectory from the Y-maze spontaneous alternation test. **c** Representative swim path from the Morris water maze (MWM) test. **d** Nanoflow cytometry analysis showing the percentage of p-tau217^+^ EVs in plasma. **e** Quantification of the NOR discrimination index. *n* = 10 in each group. **f** Quantification of spontaneous alternation behavior in the Y-maze. *n* = 10 in each group. **g** Quantification of the escape latency during the training period in the MWM (left), escape latency on day 5 of training (middle), and the time in the target platform quadrant during the test period (day 6) (right). *n* = 10 in each group. **h** Quantification of the percentage of p-tau217^+^ EVs in mice plasma. *n* = 10 in each group. **i** Colocalization of Aβ and SNAP29 shown by immunofluorescence in hippocampus. Scale bars: 100 µm. **j** Colocalization of HSPA8 and SNAP29 shown by immunofluorescence in hippocampus. Nuclei were stained with DAPI. Scale bars: 100 µm. **k** Quantification of the colocalization area between Aβ and SNAP29. **l** Quantification of the colocalization area between HSPA8 and SNAP29. *n* = 5 in each group. All data are presented as means ± SEM. ^*^P < 0.05, ^**^P < 0.01. ns, no significance

To further investigate the role of HSPA8 in p-tau217^+^ EV synthesis/secretion and AD progression, we stereotaxically injected sh-HSPA8 into the hippocampus of 6-month-old APP/PS1 mice. One month after injection, the novel object recognition test revealed no significant differences among the sh-HSPA8, sh-CTL, and WT group (Fig. 6a, e). However, the sh-HSPA8-treated APP/PS1 mice showed improved spontaneous alternation in the Y-maze (Fig. 6b, f), a significantly reduced escape latency on day 5 and increased average time spent on the platform in the water maze (Fig. 6c, g). These results indicate that HSPA8 knockdown alleviates cognitive deficits in APP/PS1 mice.

We next analyzed the proportion of p-tau217^+^ EVs in mouse plasma using nanoflow cytometry. The proportion of p-tau217^+^ EVs in plasma showed a reducing trend in APP/PS1 mice, which was reversed following HSPA8 knockdown (Fig. 6d, h). Histological assessment revealed a significant reduction in the colocalization of Aβ and SNAP29 in the sh-HSPA8 group compared to the sh-CTL group (Fig. 6i, k), accompanied by a marked increase in the colocalization of HSPA8 and SNAP29 (Fig. 6j, l). These findings suggest that HSPA8 inhibition not only improves cognitive dysfunction in APP/PS1 mice, but also restores the synthesis and secretion of p-tau217^+^ EVs.

### HSPA8-targeting peptide alleviates AD pathology and restores p-tau217^+^ EV secretion

**Fig. 7:**
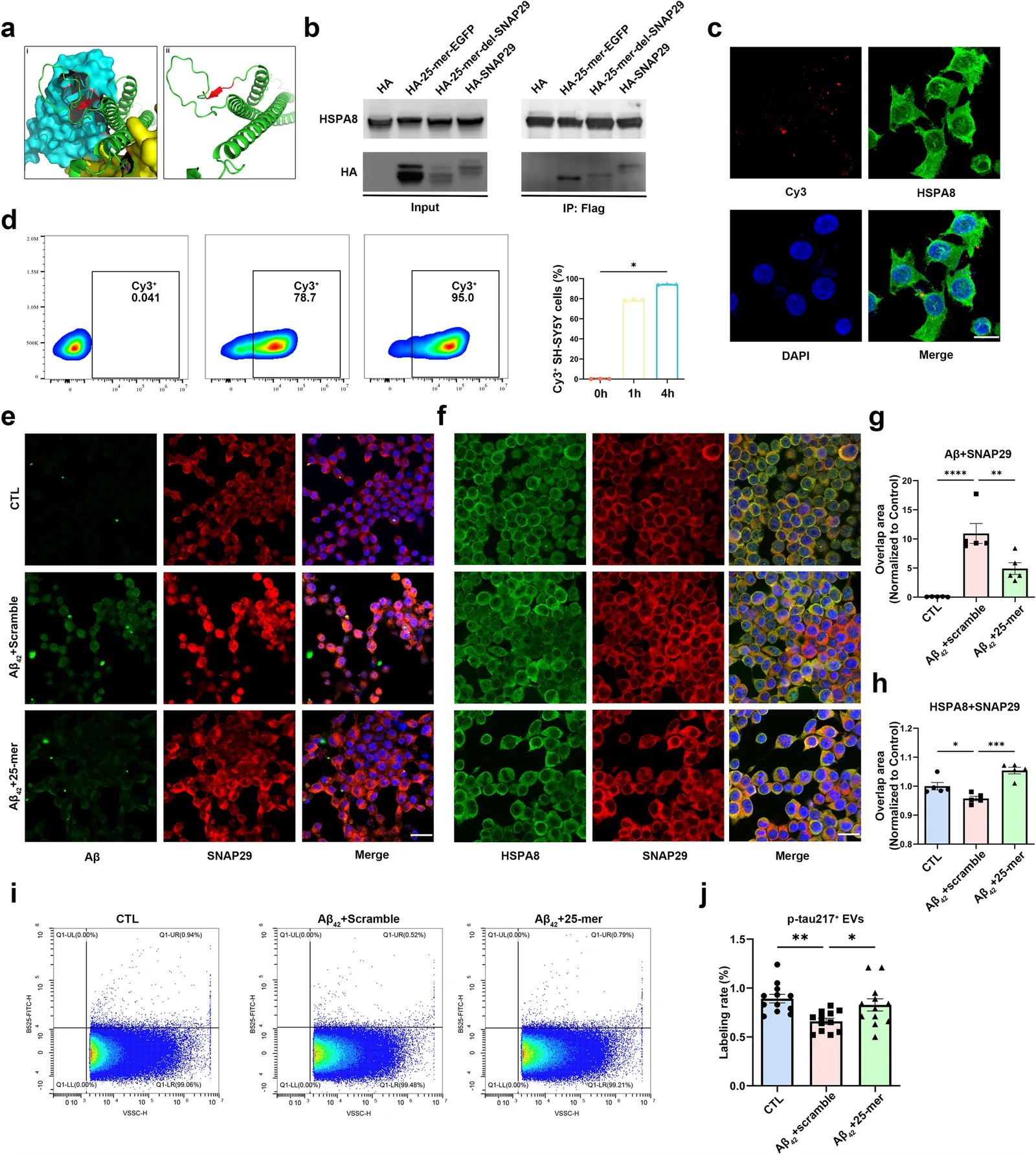
The 25-mer peptide restores p-tau217^**+**^ EV secretion in vitro. **a** The three-dimensional structure of the binding site cavity of HSPA8 bound to SNAP29 (left) and its higher magnification image (right). **b** Protein expressions of HSPA8 and HA in HEK293 cells (left), and their interaction between HSPA8 and HA (right). **c** Typical immunofluorescence images for Cy3-labeled 25-mer peptide in SH-SY5Y cells. Nuclei were stained with DAPI. Scale bars: 25 µm. **d** Flow cytometry analysis showing the uptake level of Cy3-25-mer in SH-SY5Y cells. *n* = 3 in each group. **e** Colocalization of Aβ and SNAP29 shown by immunofluorescence in SH-SY5Y cells treated with 5 μM Aβ_42_. Scale bars: 25 µm. **f** Colocalization of HSPA8 and SNAP29 shown by immunofluorescence in SH-SY5Y cells treated with 5 μM Aβ_42_. Scale bars: 25 µm. **g** Quantification of the Aβ-SNAP29 colocalization area. *n* = 5 in each group. **h** Quantification of the HSPA8-SNAP29 colocalization area. *n* = 5 in each group. **i** Nanoflow cytometry analysis showing the level of p-tau217^+^ EVs in SH-SY5Y cells. **j** Quantification of the percentage of p-tau217^+^ EVs. *n* = 12 tests in each group. All data are presented as means ± SEM. ^*^P < 0.05, ^**^P < 0.01, ^***^P < 0.001, ^****^P﻿ < 0.0001

We further designed and synthesized a small peptide to ameliorate AD pathology by modulating the function of HSPA8 in SNAP29-mediated SNARE complex assembly and p-tau217^+^ EV synthesis and secretion. We predicted the structural interaction sites between HSPA8 and SNAP29, identifying a significant binding region located within residues 166–170 of SNAP29 (Fig. 7a). Then, we synthesized a SNAP29 156–180 peptide (25-mer peptide) to block the regulatory effect of HSPA8 on Aβ–SNAP29 interaction. Co-IP in HEK293T cells confirmed direct binding between the 25-mer peptide and HSPA8 (Fig. 7b). After incubation of SH-SY5Y cells with the synthesized Cy3-labeled 25-mer peptide, immunofluorescence staining showed that the 25-mer peptide was internalized by neurons and co-localized with HSPA8 (Fig. 7c). Flow cytometry results further confirmed that the peptide was internalized by SH-SY5Y cells (Fig. 7d).

Further investigations showed that the 25-mer peptide reduced Aβ–SNAP29 colocalization in Aβ_42_-treated neurons (Fig. 7e, g) while enhancing HSPA8–SNAP29 colocalization (Fig. 7f, h). Additionally, 25-mer peptide treatment significantly increased the proportion of p-tau217⁺ EVs in the culture medium (Fig. 7i, j).

**Fig. 8:**
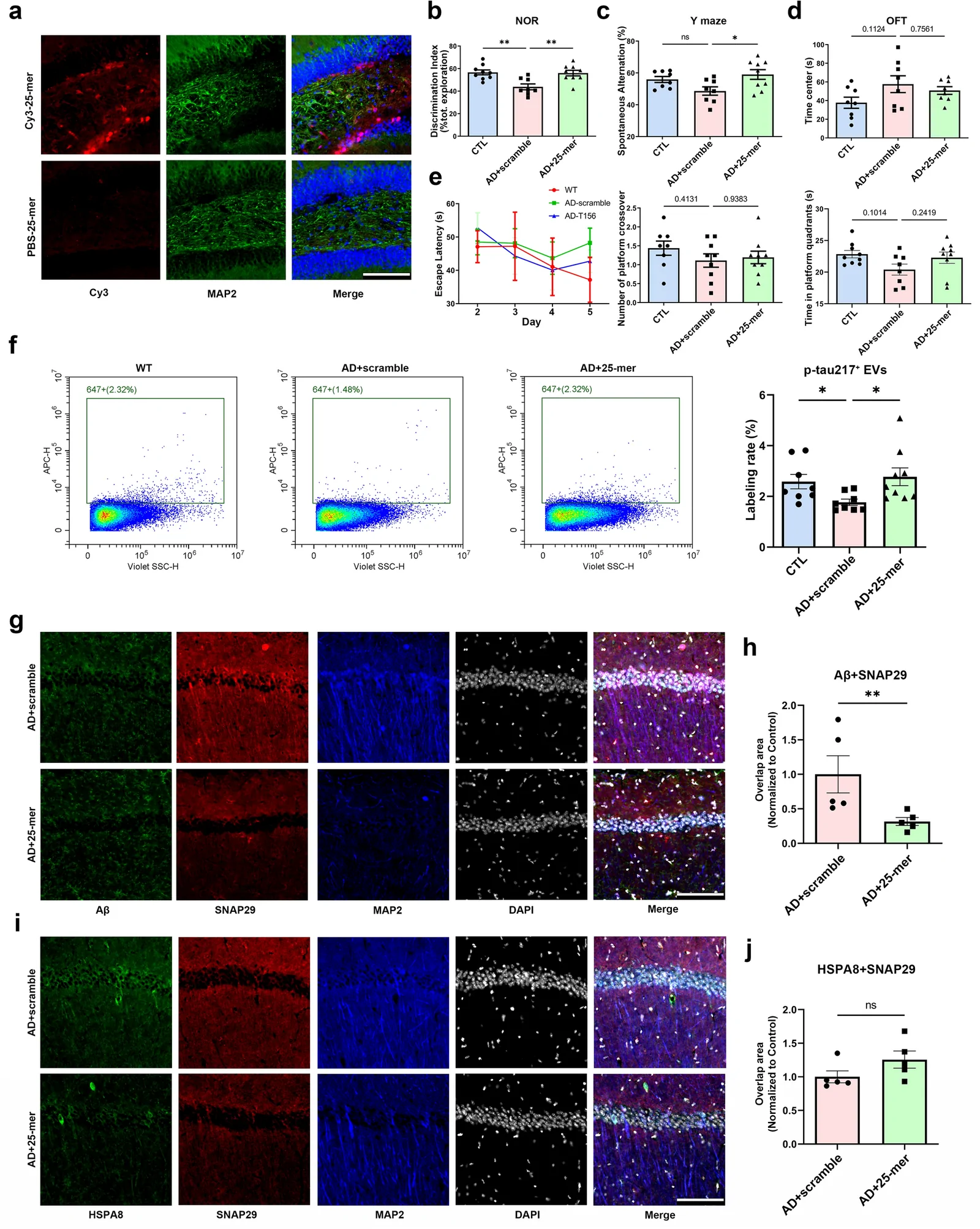
The SNAP29 25-mer peptide ameliorates cognitive deficits and restores p-tau217^**+**^ EV secretion in APP/PS1 mice. **a** Representative immunohistochemical images of hippocampal neurons stained with MAP2 (green) and Cy3-labeled 25-mer peptide (red). Nuclei were stained with DAPI. Scale bar: 100 µm. **b** Quantification of the discrimination index in NOR test. *n* = 10 in each group. **c** Quantification of spontaneous alternation behavior in Y-maze test. *n* = 10 in each group. **d** Quantification of the central time in open field test. *n* = 10 in each group. **e** Quantification of the escape latency during the training period in the MWM (left), the number of platform crossings (middle), and the time of target platform quadrants during the probe test period (right). *n* = 10 in each group. **f** Nanoflow cytometry analysis showing the percentage of p-tau217^+^ EVs in plasma. *n* = 8 in each group. **g** Colocalization of Aβ and SNAP29 shown by immunofluorescence in hippocampus. Scale bar: 100 µm. **h** Quantification of the colocalization area between Aβ and SNAP29. *n* = 5 in each group. **i** Colocalization of HSPA8 and SNAP29 shown by immunofluorescence in hippocampus. Scale bar: 100 µm. **j** Quantification of the colocalization area between HSPA8 and SNAP29. *n* = 5 in each group. All data are presented as means ± SEM. ^*^P < 0.05, ^**^P﻿ < 0.01. ns, no significance

Subsequently, we stereotaxically injected the 25-mer peptide into the hippocampus of 8-month-old APP/PS1 mice to investigate its effects in vivo. The results showed that the Cy3-labeled 25-mer peptide was effectively internalized by hippocampal cells (Fig. 8a). To explore its physiological function, unlabeled 25-mer or scrambled peptide was then administered. After 7 days of injection, the 25-mer peptide-treated mice showed improved novel object recognition ability (Fig. 8b), increased spontaneous alternation (Fig. 8c), and a trend toward reduced time in the center platform in the open field test, compared to the scramble group (Fig. 8d). Moreover, Water maze results showed an increasing trend in the number of platform crossings and the average time spent on the platform in the 25-mer peptide group (Fig. 8e).

We next assessed the effects of the 25-mer treatment on AD-related pathological changes. No significant differences were observed in Aβ plaque burden or t-tau levels between the two groups (Fig. S8a-e). In contrast, a significant reduction in the p-tau217/t-tau ratio was detected in CA1 neurons of the hippocampus in 25-mer-treated APP/PS1 mice (Fig. S8d, f), indicating an improvement in tau pathology. Then we assessed the proportion of p-tau217^+^ EVs in plasma, and found that the 25-mer peptide significantly increased the proportion of p-tau217^+^ EVs compared to the scramble group (Fig. 8f). Histological analysis revealed that the 25-mer peptide significantly reduced Aβ–SNAP29 colocalization in the hippocampus of APP/PS1 mice (Fig. 8g, h), while HSPA8–SNAP29 colocalization showed an increasing trend (Fig. 8i, j).

These results suggest that HSPA8 regulates the interaction between SNAP29 and Aβ. Disrupting the SNAP29–Aβ interaction by targeting HSPA8 with a 25-mer peptide improves cognitive deficits and restores the synthesis and secretion of p-tau217^+^ EVs in AD mice.

## Discussion

The present study primarily identifies HSPA8 as a crucial molecule in EV-mediated tau synthesis and secretion, linking vesicular trafficking machinery to the regulation of p-tau217^+^ EV release. Mechanistically, elevated HSPA8 in AD promotes SNAP29–Aβ interactions, thereby disrupting   SNARE complex assembly and impairing EV secretion. This results in intracellular accumulation of p-tau217. Inhibition of aberrant HSPA8 restores the release of p-tau217^+^ EVs, reduces neuronal tau burden, and ameliorates AD pathology, highlighting a novel potential strategy for AD therapy.

While elevated plasma free-form p-tau217 is a promising AD biomarker, our prior study revealed a decreased proportion of plasma p-tau217^+^ EVs in AD patients [18], suggesting an inverse relationship between free-form and EV-associated p-tau217. To explore the molecular mechanism underlying this phenomenon, we screened patients with CSF samples from our previously established cohort and measured free-form p-tau217 in the CSF, along with plasma from HC and AD patients, confirming the opposite directions of the two forms of p-tau217 (Fig. S1). Additionally, the opposite trend between free-form p-tau217 and p-tau217^+^ EVs was again demonstrated in the plasma of WT and APP/PS1 mice (Fig. 1i-k).

To address this intriguing phenomenon, we proposed three hypotheses: (1) transportation of p-tau217^+^ EVs from CNS to periphery is impaired in AD, causing excessive accumulation of p-tau217 in the brain; (2) the degradation of p-tau217^+^ EVs increases in AD, resulting in decreased efflux of p-tau217^+^ EVs into the peripheral blood; and (3) the synthesis and/or secretion of p-tau217^+^ EVs is impaired in AD, leading to decreased efflux of p-tau217 into the peripheral blood in the form of EVs, thereby causing excessive accumulation of p-tau217 in the brain.

To explore these hypotheses, we began with establishing an in vitro BBB model to assess the ability of p-tau217^+^ EVs to cross the BBB. Our results indicated that the equivalent amounts of p-tau217^+^ EVs, the proportion crossing the BBB model under Aβ_42_-treated conditions was significantly higher than that in the intact BBB model (Fig. S2). This finding essentially ruled out the possibility that the decreased proportion of p-tau217^+^ EVs detected in peripheral blood in AD was due to their weakened ability to cross the BBB. Furthermore, we investigated changes in cellular degradation and EV synthesis in AD. We found that lysosomal degradation was significantly reduced in Aβ_42_-treated cells, while the synthesis and secretion of EVs were even more significantly decreased (Fig. 1a, b). These findings suggest that the impaired synthesis and secretion of neuronal p-tau217^+^ EVs in AD are likely the primary cause of the opposite trends observed in the levels of free-form and EV-associated forms of p-tau217 in peripheral blood.

To further elucidate this phenomenon, mass spectrometry analysis of AD and HC CSF revealed several promising candidate molecules involved in EV synthesis or release. Considering our transcriptomics data and several in vitro and in vivo experiments, we initially focused on HSPA8. Several previous studies have reported that HSPA8 is enriched in EVs and is consistently identified across EV proteomic datasets derived from CSF, brain tissue, and plasma in AD, supporting its relevance in EV biology [34–36]. In line with these observations, our data also identified HSPA8 as a prominent component within CSF-derived EVs. However, it is important to note that our proteomic analysis was performed on immunocaptured p-tau217⁺ EVs, representing a specific EV subpopulation rather than the bulk EVs analyzed in most previous studies, suggesting that the enrichment of HSPA8 in our dataset may reflect a more specialized role in the regulation of disease-relevant EV subsets. In addition, previous studies have also shown that the expression of HSPA8 increases in AD, and knockout of HSPA8 significantly promotes axonal growth in neurons [37]. Our present study confirmed that the expression of HSPA8 was significantly elevated in AD, accompanied by the accumulation of intracellular p-tau217, along with decreased secretion of p-tau217-associated EVs.

MVBs are essential organelles for EV biogenesis and have been increasingly implicated in diverse pathophysiological processes [38]. Notably, the SNARE complex serves as a key regulatory machinery for MVB functions by mediating EV synthesis and secretion, as well as regulating subsequent EV delivery and fusion with target membranes [32]. SNAP29, a member of the SNARE complex, is involved in multiple membrane fusion processes within cells, such as endocytosis and MVB-mediated EV recycling [39]. Certain components of the SNARE complex can interact with Aβ monomers and oligomers, thereby impeding the formation of SNARE complexes and the exocytosis process [40]. Our study revealed that aberrantly elevated HSPA8 in AD exacerbates SNAP29-Aβ binding, likely disrupting the formation of functional SNARE complexes, thereby impairing p-tau217^+^ EV trafficking. Consequently, we observed that the interaction between HSPA8 and SNAP29 is diminished in AD, despite increased protein levels of both molecules. This is consistent with previous reports by Margiotta et al. [40] showing that while some SNARE complex components are elevated in AD, the formation of functional SNARE complexes is actually reduced. Notably, while our in vivo findings are largely correlative, targeted manipulation of HSPA8 in cellular models provides direct evidence supporting a causal role of HSPA8 in regulating EV biogenesis and p-tau217 secretion.

To further validate the effect of HSPA8 on p-tau217^+^ EV secretion by disrupting the interaction between Aβ and SNAP29, we designed and synthesized a 25-amino acid sequence peptide within SNAP29 that specifically targets the regulatory site of HSPA8. This competitive inhibitory peptide effectively blocks Aβ binding to SNAP29, thereby restoring the function of the SNARE complex. Additionally, blocking the regulatory interaction between Aβ and SNAP29 by the 25-mer peptide significantly improved cognitive function of APP/PS1 mice and increased the proportion of p-tau217^+^ EVs in peripheral blood. These results not only indicate that the HSPA8-mediated regulatory interaction between Aβ and SNAP29 is a key target for p-tau217^+^ EV secretion in AD, but also suggest that the 25-mer peptide, due to its properties, could serve as a useful candidate for modulating p-tau217^+^ EV secretion in the treatment of AD.

It should be stressed that, although previous reports have shown that tau is present in EVs and that disease-related phosphorylated forms of tau have been identified in AD CSF EVs [41], the detection of p-tau on the surface of EVs seems counterintuitive to conventional understanding, as it is generally considered to be predominantly intracellular. In this study, p-tau was detected on the EV membrane surface without disrupting or perforating the membrane during the experimental process. Notably, following membrane permeabilization, the detected level of p-tau217 was markedly increased (Fig. S3j), indicating that p-tau217 is present in both membrane-associated and intravesicular compartments. One possible explanation is that a subset of intracellular proteins becomes externalized onto the EV surface during vesicle biogenesis. Consistent with this notion, several studies have reported the successful detection of p-tau217, p-tau231, p-tau396, Aβ_40_, Aβ_42_, and other proteins on the EV membrane surface [18, 21, 26]. While the mechanism underlying the presence of p-tau on the EV surface warrants further investigation, our research demonstrates that Aβ and p-tau proteins are indeed present on the surface of neuron-derived EVs.

There are also several additional limitations in this study. First, although we have demonstrated that abnormally elevated HSPA8 in AD causes reduced secretion of p-tau217^+^ EVs by regulating SNAP29-mediated SNARE complex, the exact mechanism by which HSPA8 promotes the binding of SNAP29 to Aβ remains unclear. Second, we used classical APP/PS1 transgenic mice rather than the tau-mutant transgenic mice for the investigation of p-tau217 secretion. Clinically, AD patients exhibit dual Aβ and tau pathology, with extensive evidence showing that Aβ deposition can directly drive tau pathology [42, 43]. Our results also indicated changes in p-tau217 levels in the Aβ_42_-treated neurons and aged APP/PS1 mice. However, future studies are required to verify the molecular mechanisms of HSPA8 regulation of SNAP29 in tau-mutant transgenic mice. Third, although intrahippocampal injection of the SNAP29 peptide demonstrates potential therapeutic efficacy for AD, more studies are needed to explore whether this small molecular peptide can cross the BBB for peripheral administration and how to enhance its neuronal targeting.

## Conclusions

In summary, we identify HSPA8 as a critical regulator of neuronal secretion of p-tau217⁺ EVs and reveal its aberrant elevation in AD neurons. Upregulated HSPA8 impairs SNARE complex formation through altered SNAP29–Aβ interactions, reducing p-tau217⁺ EV release and worsening AD pathology. Therapeutic targeting of HSPA8 restores EV secretion and improves cognition, offering a promising new avenue for AD intervention.

## Supplementary Information

**Additional file 1. Figure S1**. The levels of free-form p-tau217 in human CSF and plasma. **Figure S2**. The efflux of p-tau217^+^ EVs across the BBB in AD. **Figure S3**. Characterization of EVs. **Figure S4**. The levels of other multiple p-tau and their EVs in Aβ-treated cells. **Figure S5**. HSPA8 knockdown efficiency confirmed by WB. **Figure S6**. The interactions between SNAP23, SNAP29, and t-tau in vitro. **Figure S7**. The interactions between SNAP23, SNAP29, HSPA8, and Aβ in CA4 and DG. **Figure S8**. Effects of 25-mer treatment on Aβ pathology and tau phosphorylation in APP/PS1 mice. **Figure S9**. Uncropped Western Blot images related to **Figure 2**. **Figure S10**. Uncropped Western Blot images related to **Figure 3**. **Figure S11**. Uncropped Western Blot images related to **Figure 4**. **Figure S12**. Uncropped Western Blot images related to **Figure 7**. **Figure S13**. Uncropped Western Blot images related to **Figure S3**. **Figure S14**. Uncropped Western Blot images related to **Figure S5**. **Table S1**. Characteristics of the human CSF and plasma samples. **Table S2**. List of primer sequences. **Table S3**. List of primary antibodies. **Table S4**. Top 15 Hub Genes.

## Data Availability

The datasets analysed in the current study are available in the CNGB Nucleotide Sequence Archive (accession code: CNP0005077; https://db.cngb.org/search/project/CNP0005077/). Further information is available from the corresponding authors upon reasonable request.

## Abbreviations

AD: Alzheimer’s disease
Aβ: Amyloid-beta
CNS: Central nervous system
CSF: Cerebrospinal fluid
DEGs: Differentially expressed genes
EVs: Extracellular vesicles
HC: Healthy control
HSPA8: Heat shock protein family A member 8
PPI: Protein–protein interaction
TEER: Trans-epithelial electrical resistance
