# Supplementary material for: HSPA8 orchestrates SNARE complex assembly to drive extracellular vesicle-mediated spread of p-tau217 in Alzheimer's disease
Source: Transl Neurodegener. 2026 Aug 27;15:42. doi: 10.1186/s40035-026-00570-4 (PMC13520189; doi:10.1186/s40035-026-00570-4)
Supplement: Supplementary file 1 [file 40035_2026_570_MOESM1_ESM.docx]

**Supplementary information for**

**HSPA8 Orchestrates SNARE Complex Assembly to Drive Extracellular Vesicle-Mediated Spread of p-tau217 in Alzheimer's Disease**

Bin Xu^1, 2, 3†^, Zhen Guo^4, 5†^, Jun Chen^1^, Yazhou Xie^1^, Pan Wang^1^, Xiao Xiong^1^, Jiayi Yu^1, 5^, Zhi Xu^1, 5^, Yuan Fu^6^, Zhuoqing Lan^6^, Guoping Peng^6*^, and Jing Zhang^1, 2, 3, 7*^

Author affiliations:

^1^ Department of Pathology, The First Affiliated Hospital, Zhejiang University School of Medicine, Hangzhou, Zhejiang, 310003, China.

^2^ Nanhu Brain-computer Interface Institute, Hangzhou, Zhejiang, 310002, China.

^3^ Lingang Laboratory, Shanghai, 200120, China.

^4^ School of Nursing and Health, Henan Medical College, Zhengzhou University, Zhengzhou, Henan, 450001, China.

^5^ School of Brain Science and Brain Medicine, Zhejiang University, Hangzhou, Zhejiang, 310012, China.

^6^ Department of Neurology, The First Affiliated Hospital, Zhejiang University School of Medicine, Hangzhou, Zhejiang, 310003, China.

^7^ National Human Brain Bank for Health and Disease, Zhejiang University, Hangzhou, Zhejiang, 310012, China.

^†^ These authors contributed equally to this work.

^*^ Correspondence

Jing Zhang, Department of Pathology, The First Affiliated Hospital, Zhejiang University School of Medicine, Hangzhou, 310003, China.

Email: jzhang1989@zju.edu.cn

Or

Guoping Peng, Department of Neurology, The First Affiliated Hospital, Zhejiang University School of Medicine, Hangzhou, 310003, China.

Email: guopingpeng@zju.edu.cn

**Figure S1. The levels of free-form p-tau217 in human CSF and plasma.** **a** The level of free-form p-tau217 in human CSF. *n* = 22 for HC and 21 for AD. **b** The level of free-form p-tau217 in human plasma. *n* = 30 for HC and 30 for AD. All values are Means ± S.E.M. ^***^*P* < 0.001, ^****^ *P* < 0.0001.

**Figure S2. The efflux of p-tau217^+^ EVs across the BBB in AD. a** Immunofluorescence images of primary cultured mouse astrocytes (GFAP), brain microvascular endothelial cells (vWF), and pericytes (NG2). Nuclei were stained with DAPI. Scale bars: 100 µm. **b** TEER value monitoring of the *in vitro* BBB model. *n* = 3 for Blank group and 6 for EPA group. TEER values were measured three times per day and averaged. **c** Nanoflow cytometry analysis showing the percentage of p-tau217^+^ EVs crossing the intact BBB model and the Aβ_42_-treated BBB model. **d** Quantification of the percentage of p-tau217^+^ EVs crossing the intact BBB and the Aβ_42_-treated BBB model. *n* = 5 in each group. All values are Means ± S.E.M. ^**^ *P* < 0.01. Abbreviation: EPA, endothelial-pericyte-astrocyte.

**Figure S3. Characterization of EVs. a** Schematic diagram of the experimental procedure. **b** NTA showed particle size and concentration of EVs extracted from the culture medium of SH-SY5Y cells. **c** Representative WB images showed the expression of CD9, TSG101, and Calnexin in isolated EVs from SH-SY5Y cells, supernatant of ultracentrifugation, and cell lysates. **d** Cryo‑EM images showing double-layered membrane‑bound vesicles in mouse plasma-derived EVs. **e** NTA showed particle size and concentration of EVs extracted from the plasma of mice. **f** Representative WB images showed the expression of CD9, TSG101, and Calnexin in isolated EVs from mouse plasma, supernatant of ultracentrifugation, and raw plasma. **g** Cryo‑EM images showing double-layered membrane‑bound vesicles in human CSF-derived EVs. **h** Cryo‑EM images showing double-layered membrane‑bound vesicles in p-tau217-immunocaptured EVs from human CSF. **i** NTA showed particle size and concentration of p-tau217-immunocaptured EVs from human CSF. **j** Nanoflow cytometry analysis showing the mean fluorescence intensity of EV-associated p-tau217 in human plasma EVs following permeabilization. *n* = 22 HC individuals. ^****^*P* < 0.0001.

**Figure S4. The levels of other multiple p-tau and their EVs in Aβ_42_-treated cells. a** Representative immunocytochemical images and quantification of the fluorescence intensity for p-tau181 in SH-SY5Y cells with or without Aβ_42_. Nuclei were stained with DAPI. Scale bar: 10 µm. **b** Representative immunocytochemical images and quantification of the fluorescence intensity for p-tau231 in SH-SY5Y cells with or without Aβ_42_. Scale bar: 10 µm. **c** Representative immunocytochemical images and quantification of the fluorescence intensity for p-tau396 in SH-SY5Y cells with or without Aβ_42_. Scale bar: 10 µm. **d** Representative immunocytochemical images and quantification of the fluorescence intensity for total tau (t-tau) in SH-SY5Y cells with or without Aβ_42_. Scale bar: 10 µm. *n* = 5 in each group. **e** Nanoflow cytometry analysis showing the percentage of p-tau181^+^ EVs in SH-SY5Y cells. **f** Nanoflow cytometry analysis showing the percentage of p-tau231^+^ EVs in SH-SY5Y cells. **g** Nanoflow cytometry analysis showing the percentage of p-tau396^+^ EVs in SH-SY5Y cells. **h** Nanoflow cytometry analysis showing the percentage of t-tau^+^ EVs in SH-SY5Y cells. *n* = 6 in each group. All values are Means ± S.E.M. ^*^*P*< 0.05, ^**^*P* < 0.01, ^***^*P* < 0.001. ns, no significance.

**Figure S5. HSPA8 knockdown efficiency confirmed by WB.** WB analysis showing the reduction of HSPA8 protein levels following RNA interference (RNAi)-mediated knockdown.

**Figure S6. The interactions between SNAP23, SNAP29, and t-tau *in vitro*.** **a** Colocalization of t-tau and SNAP23 shown by immunofluorescence (left) and quantification of the colocalization area (right). Nuclei were stained with DAPI. Scale bars: 50 µm. **b** Colocalization of t-tau and SNAP29 shown by immunofluorescence (left) and quantification of the colocalization area (right). Scale bars: 50 µm. *n* = 8 in each group. All data are presented as Means ± S.E.M. ns, no significance.

**Figure S7. The interactions between SNAP23, SNAP29, HSPA8, and Aβ in CA4 and DG. a** Colocalization of Aβ and SNAP23 shown by immunofluorescence and quantification of Aβ plaque and the colocalization area in CA4 and DG. Nuclei were stained with DAPI. Scale bars: 100 µm. **b** Colocalization of HSPA8 and SNAP23 shown by immunofluorescence and quantification of the colocalization area in CA4 and DG. Scale bars: 100 µm. **c** Colocalization of Aβ and SNAP29 shown by immunofluorescence and quantification of Aβ plaque and the colocalization area in CA4 and DG. Scale bars: 100 µm. **d** Colocalization of HSPA8 and SNAP29 shown by immunofluorescence and quantification of the colocalization area in CA4 and DG. Scale bars: 100 µm. *n* = 5 in each group. All data are presented as Means ± S.E.M. ^*^*P* < 0.05. ns, no significance.

**Figure S8. Effects of 25-mer treatment on Aβ pathology and tau phosphorylation in APP/PS1 mice. a** Representative immunofluorescence images of Aβ plaques in the hippocampus of 25-mer- or scramble-treated APP/PS1 mice. Nuclei were stained with DAPI. Scale bar: 200 µm. **b** Quantification of Aβ plaque numbers in the hippocampus. *n* = 5 in each group. **c** Quantification of area covered by Aβ plaques in the hippocampus. *n* = 5 in each group. **d** Representative immunofluorescence images of t-tau (green), p-tau217 (red) and MAP2 (blue) staining in the hippocampus. Scale bar: 50 µm. **e** Quantification of the immunofluorescence intensity for t-tau in the hippocampus. *n* = 5 in each group. **f** Quantification of the p-tau217/t-tau ratio in the hippocampus based on immunofluorescence intensity. *n* = 5 in each group. All values are Means ± S.E.M. ^*^*P* < 0.05. ns, no significance.

**Figure S9. Uncropped Western Blot images related to Figure 2.**

**Figure S10. Uncropped Western Blot images related to Figure 3.**

**Figure S11. Uncropped Western Blot images related to Figure 4.**

**Figure S12. Uncropped Western Blot images related to Figure 7.**

**Figure S13. Uncropped Western Blot images related to Figure S3.**

**Figure S14. Uncropped Western Blot images related to Figure S5.**

**Table S1.** **Characteristics of the human CSF and plasma samples**

|  | **CSF (*n* = 43)** | | **Plasma (*n* = 60)** | |
| --- | --- | --- | --- | --- |
|  | **HC** | **AD** | **HC** | **AD** |
| **Number of cases** | 22 | 21 | 30 | 30 |
| **Gender** | | | | |
| **Female** | 11 (50.0%) | 7 (33.3%) | 19 (63.3%) | 20 (66.7%) |
| **Male** | 11 (50.0%) | 14 (66.7%) | 11 (36.7%) | 10 (33.3%) |
| **Age (years)** | | | | |
| **Mean±SD** | 61.80±10.45 | 68.10±7.595 | 66.67±8.432 | 69.67±8.786 |
| **Range** | 51~77 | 53~80 | 51~79 | 57~84 |
| **CSF t-tau protein (pg/mL) (Lumipulse)** | | | | |
| **Mean±SD** | 280.4±136.0 | 433.8±274.9 | 268.9±118.2 | 475.5±219.8 |
| **Range** | 107.0~460.0 | 167.0~1140 | 107.0~460.0 | 187.0~1140 |
| **CSF p-tau181 protein (pg/mL) (Lumipulse)** | | | | |
| **Mean±SD** | 28.31±13.14 | 67.28±48.47 | 31.07±12.29 | 78.81±38.52 |
| **Range** | 11.00~51.00 | 14.00~203.0 | 14~51 | 27~103.0 |
| **CSF t-tau/Aβ_42_ (Lumipulse)** | | | | |
| **Mean±SD** | 0.7324±0.3452 | 2.683±1.810 | 0.9396±0.5510 | 3.420±2.143 |
| **Range** | 0.3920~1.411 | 0.3477~7.048 | 0.4150~2.150 | 1.363~10.00 |
| **CSF p-tau181/Aβ_42_ (Lumipulse)** | | | | |
| **Mean±SD** | 0.07117±0.02086 | 0.4153±0.3046 | 0.1189±0.09738 | 0.5643±0.3517 |
| **Range** | 0.04612~0.1144 | 0.05263~1.015 | 0.04612~0.3643 | 0.2000~1.449 |
| **CSF Aβ_42_/Aβ_40_ (Lumipulse)** | | | | |
| **Mean±SD** | 0.1216±0.02603 | 0.08142±0.03333 | 0.1074±0.04411 | 0.06148±0.02197 |
| **Range** | 0.07150~0.1610 | 0.04531~0.1619 | 0.03571~0.1810 | 0.03191~0.1389 |

Note: CSF and plasma samples were collected between 7:00 and 7:30 AM under fasting state.

Abbreviations: HC, healthy control; AD, Alzheimer’s disease; SD, Standard Deviation; CSF, cerebrospinal fluid; t-tau, total-tau; Aβ_40_, amyloid beta 1-40; Aβ_42_, amyloid beta 1-42.

**Table S2.** **List of primer sequences**

| ***Gene*** | ***Forward*** | ***Reverse*** |
| --- | --- | --- |
| *VTN* | *TGGCTGTCCTTGTTCTCCAGTG* | *GTGTGCGAAGATTGACTCGGTAG* |
| *APOE* | *GGGTCGCTTTTGGGATTACCTG* | *CAACTCCTTCATGGTCTCGTCC* |
| *VWF* | *CCTTGAATCCCAGTGACCCTGA* | *GGTTCCGAGATGTCCTCCACAT* |
| *CLU* | *TGCGGATGAAGGACCAGTGTGA* | *TTTCCTGGTCAACCTCTCAGCG* |
| *C3* | *GTGGAAATCCGAGCCGTTCTCT* | *GATGGTTACGGTCTGCTGGTGA* |
| *A2M* | *GTTGAAGAGCCTCACACGGAGA* | *TTCCACTCGGTGATGGTGTCAG* |
| *ALB* | *GATGAGATGCCTGCTGACTTGC* | *CACGACAGAGTAATCAGGATGCC* |
| *PSMA3* | *GTTTGTTGGCAGATGCTCGTTCT* | *GCAGCCAAAAGGTCTAACAGCAC* |
| *HSPA8* | *TCCTACCAAGCAGACACAGACC* | *CAGGAGGTATGCCTGTGAGTTC* |
| *AFM* | *GAACGAGATGCTGACCCAGACA* | *GGAGGGTTTTCTGTGTTGCAGC* |
| *A1BG* | *GCAACTACAGGTGCCGCTACC* | *GGCACTTCTGAGGACACCAACA* |
| *GC* | *CTGTGAGGCGTTCAGGAAAGATC* | *GTACAGCAGGACCCTACCATAG* |
| *AMBP* | *CCGACAACATCCAAGTGCAGGA* | *GTGCTCACTGTCATCCTGTCCA* |
| *SERPINC1* | *GAGACCTACCAGGACATCAGTG* | *CCTTCGGTCTTATTGGACACCC* |
| *NCAM1* | *CATCACCTGGAGGACTTCTACC* | *CAGTGTACTGGATGCTCTTCAGG* |
| *NRXN1* | *GCTATCTTGGCAGGTCCTGTGA* | *ACATCCTCAGCCTCCGTATGCA* |
| *CDH2* | *CCTCCAGAGTTTACTGCCATGAC* | *GTAGGATCTCCGCCACTGATTC* |
| *SNAP23* | *GGTGACAAATGGTCAGCTTCAGC* | *CCAGGATACTGCCCACTTGAGT* |
| *SNAP25* | *CGTCGTATGCTGCAACTGGTTG* | *GGTTCATGCCTTCTTCGACACG* |
| *SNAP29* | *CAGCCACCCAAACCTTAGAAAGC* | *CGATCTTCTGGTGATAGGCTCG* |
| *SNAP47* | *AGAAGAGCTGCTCAGTCTGGCA* | *AGTTCCTGGGTATCTGCCTCAG* |
| *VAMP2* | *CTCCAAACCTCACCAGTAACAGG* | *AGCTCCGACAGCTTCTGGTCTC* |
| *VAMP3* | *GCTCTCTGAGTTAGACGACCGT* | *CCAGAACAGTAATCCCGATTGCC* |
| *VAMP4* | *GCTTATCGGATAATGCAACAGCTT* | *GCAGCAACCAAAGCCATGATGG* |
| *VAMP7* | *CGGTTCAAGAGCACAGACAGCA* | *ATCCACTTGGGCTTGAGTCTCC* |
| *VAMP8* | *TATGACCCAGAATGTGGAGCGG* | *ATTTCCGAGCCACCTTCTGCGA* |
| *YKT6* | *CTGTTAGAGCGAGGTGAGAAGC* | *GATGGTGCCATTCCAGCATTGG* |
| *STX3* | *TCATCGGCAGACCTTCGGATTC* | *CTTTGCTGCGTTCTCGGAAGTC* |
| *STX4* | *CGGCAGACTATTGTCAAACTGGG* | *ATCTCATCGCGCAGGTTCTGCA* |
| *STX11* | *GAGATGAAGCAGCGCGACAACT* | *CCAGCAAGTTCTCGGAAAACACG* |
| *STX16* | *AGCTGTTAGCCGAGCAAGTGAG* | *GCCGTTTTGTCACACCAATCGC* |
| *STX18* | *GGAGGATGACAGACACAGAACG* | *GCTCCTTCACTTGCTGGGAATG* |
| *GAPDH* | *GTCTCCTCTGACTTCAACAGCG* | *ACCACCCTGTTGCTGTAGCCAA* |

**Table S3. List of primary antibodies**

| **Application** | **Antibody** | **Supplier** | **Catalog number** | **RRID** | **Dilution** |
| --- | --- | --- | --- | --- | --- |
| WB | p-tau217 | Thermo | 44744 | AB_2533741 | 1:500 |
|  | HSPA8 | Abcam | ab51052 | AB_880538 | 1:500 |
|  | SNAP23 | Proteintech | 10825-1-AP | AB_2192022 | 1:1000 |
|  | SNAP25 | Proteintech | 14903-1-AP | AB_2192051 | 1:1000 |
|  | SNAP29 | Proteintech | 12704-1-AP | AB_2192340 | 1:500 |
|  | VAMP7 | Proteintech | 22268-1-AP | AB_11182718 | 1:2000 |
|  | VAMP8 | Thermo | MA5-32502 | AB_2809779 | 1:1000 |
|  | STX4 | Proteintech | 68429-1-Ig | AB_3085143 | 1:5000 |
|  | STX16 | Proteintech | 66775-1-Ig | AB_2882121 | 1:2000 |
|  | β-Amyloid_1-42_ | BioLegend | 805501 | AB_2564683 | 1:500 |
|  | HA Tag | Thermo | 26183 | AB_10978021 | 1:5000 |
|  | TSG101 | Proteintech | 67381-1-Ig | AB_2882628 | 1:5000 |
|  | CD9 | Proteintech | 60232-1-Ig | AB_11232215 | 1:5000 |
|  | Calnexin | Proteintech | 66903-1-Ig | AB_2882231 | 1:10000 |
|  | β-actin | Proteintech | 66009-1-Ig | AB_2687938 | 1:500 |
| IF | HSPA8 | Proteintech | 66442-1-Ig | AB_2881812 | 1:200 |
|  | SNAP23 | Proteintech | 10825-1-AP | AB_2192022 | 1:200 |
|  | SNAP29 | Proteintech | 12704-1-AP | AB_2192340 | 1:200 |
|  | MAP2 | Abcam | ab5392 | AB_2138153 | 1:10000 |
|  | β-Amyloid_1-42_ | BioLegend | 805501 | AB_2564683 | 1:500 |
|  | GFAP | Abcam | ab218309 | AB_2909614 | 1:800 |
|  | vWF | Abcam | ab6994 | AB_305689 | 1:800 |
|  | NG2 | Abcam | ab129051 | AB_2877152 | 1:800 |
|  | p-tau217 | Thermo | 44744 | AB_2533741 | 1:200 |
|  | p-tau181 | Thermo | MN1050 | AB_223651 | 1:200 |
|  | p-tau231 | Thermo | MN1040 | AB_223649 | 1:200 |
|  | p-tau396 | Thermo | 35-5300 | AB_2533211 | 1:200 |
|  | t-tau | Thermo | MA5-12808 | AB_10980631 | 1:200 |
|  | synaptophysin | Sigma-Aldrich | SAB4502906 | AB_10746692 | 1:200 |
|  | CD9 | Proteintech | 60232-1-Ig | AB_11232215 | 1:400 |

**Table S4. Top 15 Hub Genes**

| **Rank** | **Name** | **Score** |
| --- | --- | --- |
| 1 | *ALB* | 43 |
| 2 | *GAPDH* | 42 |
| 3 | *VTN* | 25 |
| 4 | *HSPA8* | 24 |
| 5 | *APOE* | 22 |
| 6 | *C3* | 21 |
| 7 | *A2M* | 21 |
| 8 | *PSMA3* | 20 |
| 9 | *CLU* | 20 |
| 10 | *VWF* | 19 |
| 11 | *CDH2* | 15 |
| 12 | *GC* | 13 |
| 13 | *AFM* | 13 |
| 14 | *AMBP* | 13 |
| 15 | *A1BG* | 12 |
| 15 | *NCAM1* | 12 |
| 15 | *SERPINC1* | 12 |
| 15 | *NRXN1* | 12 |
